# Engineering Nanoscale Frontiers: Valve Metal Oxide Nanostructures From Fundamentals to Multifunctional Biomedical Applications

**DOI:** 10.1002/tcr.70185

**Published:** 2026-05-28

**Authors:** Nina Kummer, Désirée Gül, İdris Sargin, Mustafa Dolaz, Dzmitry Shcharbin, Shirley K. Knauer, Burcu Önal Acet, Emrah Dikici, Mingyuan Gao, Mehmet Odabaşı, Ömür Acet, Roland H. Stauber

**Affiliations:** ^1^ Nanobiomedicine/Molecular and Cellular Oncology ENT University Medical Center Mainz Mainz Germany; ^2^ Department of Biochemistry Selçuk University Konya Turkey; ^3^ Department of Environmental Engineering Faculty of Engineering Kyrgyz‐Turkish Manas University Bishkek Kyrgyz Republic; ^4^ Department of Environmental Engineering Faculty of Engineering and Architecture Kahramanmaras Sutcu Imam University Kahramanmaras Turkey; ^5^ Institute of Biophysics and Cell Engineering of the National Academy of Sciences of Belarus Minsk Belarus; ^6^ Department of Molecular Biology II Center for Medical Biotechnology (ZMB)/Center for Nanointegration Duisburg‐Essen (CENIDE) University of Duisburg‐Essen Essen Germany; ^7^ Department of Engineering Fundamental Sciences Faculty of Engineering Artvin Çoruh University Artvin Turkey; ^8^ Medicinal‐Aromatic Plants Application and Research Center Artvin Çoruh University Artvin Turkey; ^9^ Dogubayazit Ahmed‐i Hani Vocational School Agri Ibrahim Cecen University Agri Turkey; ^10^ Central Researching Laboratory Agri Ibrahim Cecen University Agri Turkey; ^11^ School of Life Sciences Suzhou Medical College Soochow University Suzhou China; ^12^ Faculty of Arts and Science Chemistry Department Aksaray University Aksaray Turkey; ^13^ Vocational School of Health Science Pharmacy Services Program Tarsus University Tarsus Mersin Turkey

**Keywords:** biocompatible nanomaterials, biomedical applications, nanostructured metal oxides, surface functionalization, valve metal oxides

## Abstract

Valve metal oxide nanostructures represent a frontier in biomaterial science, where deliberate surface engineering at the nanoscale directly dictates their biological performance. This synergy enables the creation of multifunctional platforms capable of addressing complex challenges in diagnostics, therapy, and regenerative medicine. TiO_2_, Ta_2_O_5_, Nb_2_O_5_, ZrO_2_, and HfO_2_ constitute a remarkable class of valve metal oxide nanostructures that combine exceptional stability, tunable surface properties, and biocompatibility. Their intrinsic mechanical robustness and corrosion resistance are critically important for biomedical implants, providing the essential structural integrity required for long‐term in vivo performance and osseointegration. Their nanometer‐scale architectures facilitate a wide range of biomedical applications, from implant coatings and antimicrobial surfaces to drug delivery, biosensors, and phototherapy. Furthermore, their photocatalytic and piezoelectric properties expand their potential as versatile tools for next‐generation therapeutic strategies. Due to their future potential, these robust materials are also indispensable for personalized and targeted medicine. This review details the fundamental synthesis techniques, physicochemical properties, and biological interactions of valve metal oxides, highlighting their importance in enhancing biofunctionality and therapeutic efficacy. Current challenges regarding safety, scalability, and clinical application are also examined, highlighting their potential roles as multifunctional platforms for biomedical advancement.

## Graphical abstract

Valve metal oxide nanostructures such as TiO_2_, Ta_2_O_5_, Nb_2_O_5_, ZrO_2_, and HfO_2_ are emerging as versatile biomedical platforms due to their tunable surface properties, exceptional stability, and inherent biocompatibility. This review highlights their synthesis, physicochemical properties, and biological interactions, addressing their roles in advanced implants, drug delivery, biosensors, and next‐generation therapeutic applications.

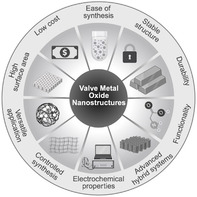

## 1 Introduction

Nanotechnology holds significant potential in many fields [1]. Today, advances in materials science have played a crucial role in creating innovative nanoscale systems, particularly for various biomedical applications [2, 3, 4, 5].

The growing use of metal/metal oxide nanoparticle‐polymer hybrid systems for biochemical applications can be attributed to their exceptionally tunable porosities, large surface areas, and diverse functional properties. These innovative materials exhibit superior biocompatibility, antibacterial properties, and controlled drug release capabilities, making them highly suitable for drug delivery, medical imaging, biosensors, and tissue engineering. By integrating metals and metal oxide nanoparticles into the polymer matrix, the mechanical strength, chemical resistance, and responsiveness of mesoporous polymers are significantly enhanced, expanding their potential applications in advanced medical technologies [6, 7].

**FIGURE 1:**
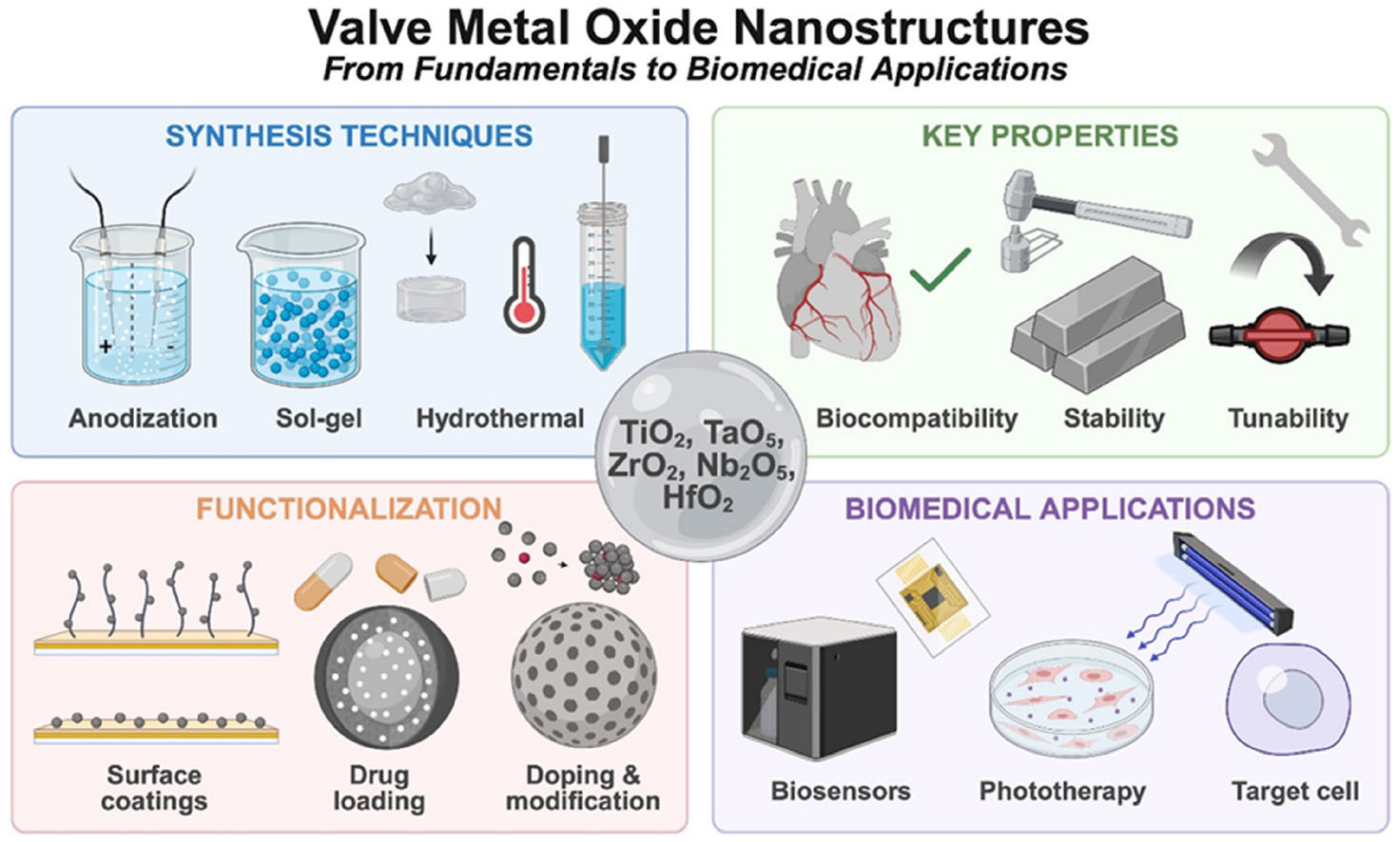
Valve metal oxide nanostructures: from fundamental to biomedical applications.

Metal oxide nanostructures represent an important category of nanomaterials highly desirable for a wide range of applications, from electronics and biomedicine to energy conversion, due to their exceptional properties and valuable functionality. Key characteristics of metal oxide nanostructures include their abundance and generally high stability. Achieving controlled growth of metal oxide nanostructures with desired size, shape, and crystal structure remains a fundamental goal in nanomaterial research, yet it also presents a significant challenge today. Extensive research efforts have been undertaken in both experimental and theoretical fields to address this goal [8, 9, 10, 11, 12]. In particular, metal oxide nanoparticles offer numerous advantages, including exceptional stability, straightforward preparation methods, and the ability to engineer them to the desired size, shape, and porosity. They exhibit no swelling variations, can be easily integrated into both hydrophobic and hydrophilic systems, and allow for effortless functionalization with various molecules due to their negatively charged surface. These attributes render them a highly promising asset for biomedical applications (Figure 1). Valve metal oxides (VMOs), notably titanium dioxide, zirconium dioxide, niobium pentoxide, and tantalum pentoxide, represent some of the most significant categories of oxides utilized in the field of biomedicine. These oxides carry out essential biomedical functions across a range of sizes [13, 14, 15, 16, 17, 18, 19].

VMO nanostructures have emerged as a crucial class of biomaterials by effectively bridging fundamental surface engineering principles with advanced biomedical applications. Their intrinsic chemical stability, tunable nanoscale architecture, and favorable biological interactions not only enable precise interface design but also allow active modulation of biological responses, rather than merely serving as passive substrates. Building on these advantages, recent progress in synthesis and surface functionalization has significantly expanded their applicability. However, despite these developments, critical challenges remain, particularly in achieving reproducible large‐scale fabrication, ensuring long‐term biosafety, and facilitating their seamless translation into clinically relevant systems. These interconnected limitations highlight the need for an integrated, multidisciplinary approach that combines materials science, surface chemistry, and biomedical engineering. In this context, the present review systematically establishes the relationship between synthesis, structure, physicochemical properties, and biointeraction behaviors of VMO nanostructures. By doing so, it provides a coherent framework to understand how these parameters collectively influence performance across biomedical applications. Specifically, the review covers fundamental material characteristics, classification and synthesis strategies, surface functionalization approaches, and their direct implications in key areas such as implant coatings, antimicrobial and wound healing systems, drug delivery, biosensing and diagnostics, phototherapy, imaging, and tissue engineering. Through this structured perspective, the review aims to guide future research toward the safe, scalable, and effective utilization of VMO nanostructures in next‐generation biomedical technologies.

Given the impressive combination of properties and multifunctional potential of VMO nanostructures, this review aims to provide a comprehensive and critical synthesis of the current state of VMO nanostructures in biomedicine. The synthesis methodologies will systematically examine the interplay between nanoscale physicochemical properties and the resulting biological interactions. A detailed analysis of surface functionalization strategies is presented to elucidate the principles of biological interface engineering specific to this class of materials. Transducer applications such as implantable device coatings, targeted therapeutic delivery systems, biosensor platforms, antimicrobial surfaces, and phototherapeutic agents are discussed in depth. Simultaneously, the review also confronts persistent translational challenges such as scalable fabrication, rigorous long‐term biosafety assessment, and seamless clinical integration that must be overcome to fully realize their promises. This study aims to integrate fundamental materials science with applied biomedical engineering perspectives to create a coherent framework that will guide future research into the rational design, safe application, and clinical implementation of these versatile nanostructures, thereby positioning them as fundamental elements of next‐generation diagnostic and therapeutic methods.

## 2 Fundamental Materials and Their Properties

**FIGURE 2:**
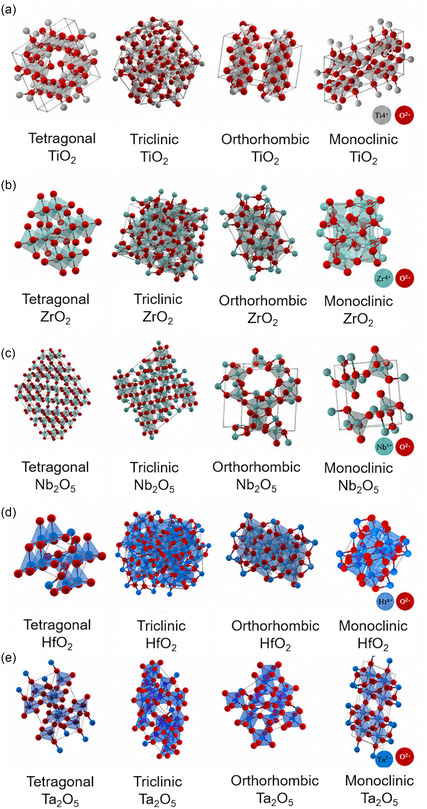
Comparative illustration of representative geometries of VMOs. (Ref: *Materials Data on WMO by Materials Project*. United States.).

VMOs—primarily titanium dioxide (TiO_2_), tantalum pentoxide (Ta_2_O_5_), niobium pentoxide (Nb_2_O_5_), zirconium dioxide (ZrO_2_), and hafnium dioxide (HfO_2_)—constitute a significant class of functional ceramics defined by their ability to form stable, self‐passivating oxide layers. This inherent property confers exceptional chemical inertness, corrosion resistance, and long‐term stability in aggressive physiological environments, making it a key criterion for their biomedical suitability. Beyond mere passivation, their value is revealed through precise nanoscale engineering that tailors their physicochemical properties—bandgap, surface energy, topography, and crystal structure—to dynamically interact with biological systems. This section provides a systematic overview of these five fundamental materials, examining the intrinsic properties that govern their biomedical performance. For each oxide, we investigate its crystallographic phases, electronic structure, and fundamental properties such as biocompatibility, mechanical strength, and optical behavior. To facilitate a better understanding of the structural diversity of VMOs, Figure 2 includes a comparative figure showing representative geometries of (a) TiO_2_, (b) ZrO_2_, (c) Nb_2_O_5_, (d) HfO_2_, and (e) Ta_2_O_5_. Understanding these fundamental material properties is crucial because they directly determine subsequent strategies for nanostructuring, surface functionalization, and ultimately, specific therapeutic or diagnostic applications, which are discussed in later sections of this review.

### 2.1 Titanium Dioxide (TiO_2_)

Titanium (IV) oxide is a form of titanium oxide typically found in three crystalline forms: rutile and anatase (both tetragonal) and brookite (orthorhombic). Among these, rutile is widely recognized for its high refractive index, its ability to scatter visible light, and its ability to absorb UV light—qualities that make it a common pigment in paints, sunscreens, and food additives, as well as for photocatalytic H_2_ evolution. Anatase, on the other hand, is valued for its photocatalytic properties, particularly in environmental remediation and solar energy applications, due to its surface defects that enhance reactivity [20].

Both rutile and anatase consist of titanium atoms coordinated by six oxygen atoms in distorted octahedral units. The degree of distortion and connectivity differs between the two phases: rutile exhibits minimal distortion and an elongated unit cell, while anatase shows greater distortion and lower symmetry. These structural variations influence the electronic band structure and photoinduced charge‐carrier behavior. Interestingly, particle size plays a role in phase stability: rutile is favored for particles larger than 35 nm, whereas anatase is more stable at the nanoscale (below 20 nm) [20, 21]. Surface area and morphology can be tailored during synthesis, which is critical for biomedical applications. In aqueous environments, TiO_2_ nanoparticles tend to agglomerate, reducing their effective surface area and photoactivity. To address this, surface modifications, such as electrostatic or steric functionalization, are often employed to stabilize dispersions and improve performance in biological systems [22].

The chemical behavior of TiO_2_ nanoparticles is strongly size‐dependent. Smaller particles exhibit higher surface reactivity and greater potential for cellular uptake. Advanced synthesis techniques allow precise control over crystal facets, enhancing catalytic and biological activity. TiO_2_ is inherently chemically and thermally stable, making it suitable for physiological environments. Its optical properties can be tuned through doping with metals (e.g., Au, Fe, Pt) or nonmetals (e.g., N, C), as well as by coupling with organic molecules such as photosensitizers or polymers [23]. These modifications extend light absorption into the visible or near‐infrared range, improving efficiency in photodynamic therapy (PDT) and antimicrobial applications [24]. Upon light activation, TiO_2_ generates reactive oxygen species (ROS), including superoxide, hydrogen peroxide, and hydroxyl radicals, which are cytotoxic to cancer cells and pathogens [25]. This photocatalytic activity underpins its role in PDT, antimicrobial coatings, and sterilization processes.

TiO_2_ is generally considered biocompatible and is widely used in medical implants, dental materials, and drug delivery systems. Its inert nature minimizes adverse reactions, while surface functionalization with biomolecules enables targeted therapies. For example, Venkatasubbu et al. [26] developed folic acid‐targeted, polyethylene glycol (PEG)‐coated TiO_2_ nanoparticles as carriers for paclitaxel. In that study, the TiO_2_ nanoparticles were coated with PEG to avoid immune clearance by the reticuloendothelial system (RES) and enhance biocompatibility. They were also grafted with folic acid to target folate receptors, which are overexpressed in cancer cells.

Although TiO_2_ exhibits low acute toxicity, chronic exposure, particularly via inhalation, may pose risks due to tissue penetration and immune activation. Toxicity is influenced by particle size, surface chemistry, and crystal phase; anatase tends to be more phototoxic because of its higher ROS generation [27]. Despite the toxicity concerns related with TiO_2_ NPs, TiO_2_ finds applications in biomedical practice, PDT for cancer and bacterial infections, targeted drug delivery (e.g., doxorubicin‐loaded systems), wound healing, tissue engineering, and antimicrobial coatings. Its ability to integrate with bone tissue also makes it a preferred choice for orthopedic and dental implants.

### 2.2 Tantalum Pentoxide (Ta_2_O_5_)

Tantalum (V) oxide, also called tantalum pentoxide (Ta_2_O_5_), is a white, odorless compound made from tantalum and oxygen. Tantalum can be found in both +5 and +4 oxidation states, known as Ta_2_O_5_ and TaO_2_, respectively. Of these, Ta_2_O_5_ is considered the most thermodynamically stable form. It is a durable metal oxide with a very high melting point and does not dissolve in water. Tantalum pentoxide (Ta_2_O_5_) is a highly regarded VMO known for its exceptional chemical stability, corrosion resistance, and biocompatibility. It can exist in amorphous or crystalline forms, possessing a high‐dielectric constant and a wide bandgap, which make it particularly valuable for biomedical and electronic applications [28, 29, 30, 31].

Widely used in electronics, Ta_2_O_5_ is key to the manufacturing of capacitors and thin‐film devices and is used as a dielectric material because of its high permittivity and stability. As an optically transparent metal oxide with a high refractive index, it also resists corrosion well, making it useful for optical coatings and catalysts. Mainly, it acts as a high‐dielectric component in capacitors and helps with electron transport and surface passivation in perovskite solar cells [29, 30, 31]. Ta_2_O_5_ is a transition‐metal oxide with a wide bandgap, high‐dielectric constant, and tunable bandgap, making it useful in optical, dielectric, photocatalytic, electrocatalytic, and nonvolatile memory applications [28]. Its thin films are chemically stable, with low reflectivity and high refractive index, often used as antireflection coatings on silicon solar cells to boost efficiency [31, 32].

In the biomedical field, Ta_2_O_5_ coatings are commonly applied to implants and prosthetic devices to promote better integration with bone and ensure long‐term stability. Their inertness and resistance to corrosion in the body help minimize the release of ions and reduce inflammatory responses, making them more biocompatible than many traditional materials. The surface of Ta_2_O_5_ can also be engineered at a nanoscale level to support cell attachment, growth, and differentiation. This makes it especially useful for bone and dental tissue engineering. Nanostructured Ta_2_O_5_ surfaces can mimic the natural architecture of the extracellular matrix, which enhances cell interactions and accelerates tissue regeneration [33, 34].

Ta_2_O_5_ shows great promise in medicine because it's highly biocompatible, chemically stable, and corrosion‐resistant. These qualities make it ideal for applications such as implant coatings, where it can enhance surface interactions with the body and reduce the risk of immune responses [35]. Additionally, Ta_2_O_5_ has good mechanical properties and can be modified to promote cell attachment and growth, which is helpful for bone healing and dental implants [36]. As research advances, its versatile features continue to support its potential in more sophisticated medical devices and tissue repair [37, 38].

### 2.3 Niobium Pentoxide (Nb_2_O_5_)

Niobium is a transition metal that can form several oxide compounds, such as NbO, Nb_2_O_3_, NbO_2_, and Nb_2_O_5_, with niobium exhibiting oxidation states of +2, +3, +4, and +5, respectively. Among these, niobium pentoxide (Nb_2_O_5_) is the most thermodynamically stable within the niobium–oxygen system. This compound is particularly significant for its potential use in energy storage, where niobium's valence state shifts from +5 to +4 or even +3 during redox reactions [39].

Niobium pentoxide (Nb_2_O_5_) is an n‐type semiconductor with a bandgap of around 3.4 eV. Its stable structure and useful properties make it valuable for a range of technologies, including gas sensors, electrochromic devices, photoelectrochemical cells, and display or microelectronic components [40]. Niobium pentoxide is a key oxide with versatile uses in electrochromism and catalysis. It facilitates the formation of crystals such as KNbO_3_ and NaNbO_3_ for technological applications. Nontoxic Nb_2_O_5_ can also be used in water purification. Its photocatalytic efficiency improves through control over structural and morphological factors during synthesis. Technologically, niobium pentoxide can absorb ultraviolet light, making it valuable for shielding materials from UV radiation. The oxide has various structural phases—trigonal, hexagonal, monoclinic, and orthorhombic—that affect its physical properties, enabling it to act as either a semiconductor or a dielectric, depending on its symmetry [41].

Various nanoparticles are used in biomaterials, but niobium compounds have received less attention. Niobium nanoparticles are promising due to their lower biocompatibility than that of other metals in cell studies. Niobium oxide (Nb_2_O_5_), in particular, has demonstrated excellent compatibility when incorporated into fluorapatite glass ceramics and Ti–Nb_2_O_5_ metal composites [42, 43]. It also enhances biological activities, such as enzyme activity, hydroxyapatite growth, and cell spreading [44, 45]. Additionally, membranes containing niobium pentoxide improve cell metabolism [46]. These benefits have led to interest in using Nb_2_O_5_ nanoparticles for wound healing. They are typically produced via microwave‐assisted hydrothermal synthesis, a clean, efficient, and eco‐friendly method that yields small, reactive particles quickly and at low cost [47].

### 2.4 Zirconium Dioxide (ZrO_2_)

Zirconium dioxide (ZrO_2_), also known as zirconia, is a ceramic material valued for its impressive strength, toughness, and chemical stability [48]. It naturally exists in monoclinic, tetragonal, and cubic forms, but doping it with stabilizers like yttria helps preserve the stronger tetragonal or cubic phases at room temperature. This stabilization enhances its toughness by allowing stress‐induced transformations [49]. With its high hardness, wear resistance, and corrosion resistance, ZrO_2_ is widely used in load‐bearing biomedical applications [50].

Zirconia nanoparticles are highly valued for their biodegradability and remarkable electrical, mechanical, and optical properties [51]. Since the development of methods to produce nanosized particles, researchers worldwide have shown significant interest in zirconium oxide owing to its high luminous efficiency, broad bandgap, and strong exciton binding energy [52]. These attributes make ZrO_2_ NPs suitable for applications such as antimicrobial agents [53] and anticancer agents [54]. Recently, various synthesis techniques, including green chemistry approaches, have been employed to fabricate ZrO_2_ nanocomposites with diverse morphologies, reflecting ongoing scientific innovation in this area [55].

Among various materials used in biomedical applications, zirconium dioxide stands out as a promising candidate, especially for bone tissue engineering [56] and orthopedic implants [57]. The increasing challenges with implant loosening and the need for effective bone regeneration have driven more research into this ceramic. Zirconium dioxide is valued for its strong properties, including high compressive strength (around 2000 MPa), excellent support for cell growth, biocompatibility, durability against fracture, and radiopacity, and is also nontoxic to cells [58]. The use of zirconium dioxide in biomedical applications has advanced solutions for challenges in orthopedic implants and bone tissue engineering. It offers strong mechanical properties, good fracture resistance, biocompatibility, and excellent radiopacity, which improves visibility during imaging. Biomaterials containing ZrO_2_ have shown sufficient strength and compatibility for medical use. Electrochemical treatments can improve corrosion resistance, extending implant lifespan. Adding ZrO_2_ also helps create porous surfaces, promoting better cell growth, crucial for bone regeneration. Its radiopacity is especially valuable in orthopedic bone cements [58, 59, 60].

### 2.5 Hafnium Dioxide (HfO_2_)

Hafnium dioxide (HfO_2_), known as hafnium (IV) oxide, is a metal oxide that offers significant advantages in thermal stability and dielectric properties. It is well recognized for its high melting point, chemical inertness, and wide bandgap, making it suitable for biomedical and electronic applications. HfO_2_ is a stable, colorless inorganic compound with an approximate bandgap of 5.3–5.7 eV. Its high melting point (∼2800°C) and low thermal conductivity make it a refractory material. HfO_2_ nanoparticles are used in various fields, including optoelectronics, photocatalysis, scintillators, UV sensors, and biomedicine, notably as radiosensitizers in cancer radiotherapy due to their high‐dielectric constant and chemical stability [61, 62, 63]. For instance, McGinnity et al. [64] reported that HfO_2_ NPs, measuring 60–90 nm, remained stable in both water and cell culture media for several days without clumping or sedimenting. They were nontoxic to human epithelial cells and macrophages for up to 24 h at concentrations up to 0.8 mg/mL. When labeled with Cy5, the nanoparticles allowed for fluorescence imaging of cellular uptake but showed slightly increased toxicity compared to unmodified particles. Both fluorescence microscopy and TEM confirmed that the HfO_2_ NPs were taken up by the cells, with evidence of macropinocytosis and endosomal localization within 4 h. Overall, these HfO_2_ nanoparticles demonstrate stability, biocompatibility, and effective cellular entry, indicating their potential as theranostic agents in nanomedicine.

At the nanoscale, HfO_2_ surfaces can be engineered to enhance cell adhesion, proliferation, and differentiation, supporting applications in bone and dental tissue engineering. In a study by Fohlerova and Mozalev, films of nanostructured HfO_2_ showed promise for biomedical applications due to their biocompatibility, high density, and resistance to corrosion and mechanical damage [65]. The report indicates that studies involving MG‐63 osteoblast‐like cells and *E. coli* demonstrate that these films are well tolerated by cells and attract proteins, such as albumin, with a ninefold increase in efficiency. The nanostructure enhances antibacterial efficacy, facilitates improved cell attachment and proliferation, and notably increases cellular stiffness. In another study [66], HfO_2_ films deposited by atomic layer deposition (ALD) were shown to promote the formation of biological apatite. The films retained their quality while serving as effective biomaterials.

### 2.6 Design‐Oriented Selection of Valve Metal Oxides for Biomedical Applications: Performance Niches and Trade‐Offs

Taken together, these oxide systems reveal that biomedical utility emerges not from a single universal advantage, but from oxide‐specific balances between stability, reactivity, and biological interaction. From a biomedical perspective, VMOs should not be regarded as functionally interchangeable materials, but rather as platforms occupying distinct performance niches. TiO_2_ remains the most versatile system because its established nanostructuring routes, strong photocatalytic behavior, and comparatively narrow bandgap support a broad spectrum of applications ranging from antimicrobial surfaces and phototherapy to drug delivery and tissue engineering. However, this versatility is accompanied by important trade‐offs, including agglomeration‐related loss of effective surface area and greater sensitivity to phase‐ and dose‐dependent phototoxicity. Nb_2_O_5_ occupies an intermediate position between photoresponsive and bioinert oxides, offering a favorable combination of semiconducting behavior, corrosion resistance, and encouraging osteogenic and cytocompatibility profiles, although its biomedical literature remains less mature than that of TiO_2_, and its application space is still comparatively underdefined. In contrast, Ta_2_O_5_ is more appropriately viewed as a stability‐driven coating material whose primary advantages lie in chemical inertness, corrosion protection, and strong interfacial biocompatibility rather than intrinsic photoactivity. ZrO_2_ and HfO_2_ further reinforce this stability‐oriented end of the spectrum, contributing superior mechanical robustness, dielectric character, and structural durability, but generally offering less functional flexibility for photoactivated or redox‐mediated biomedical strategies. Accordingly, the selection of a given VMO should be guided not simply by biocompatibility alone, but by the specific balance required among photoresponsiveness, corrosion resistance, mechanical reliability, biological stimulation, and translational readiness [13, 14, 15, 16, 17, 18, 19, 20, 33, 34, 35, 36, 37, 38, 67].

Importantly, the synthesis strategy should be interpreted not merely as a fabrication choice, but as a decisive factor governing the final biomedical value of each VMO system. In this respect, anodization is particularly advantageous for TiO_2_, since it enables the formation of highly ordered nanotubular architectures with controllable diameter, length, and wall thickness, thereby making it especially suitable for applications in which cell guidance, surface‐mediated osteogenesis, drug loading, or photoresponsive functionality are required. However, this strong architectural control is also relatively material‐specific and may not be transferred with equal efficiency to all VMO systems. By comparison, sol–gel and hydrothermal/solvothermal methods provide broader flexibility in tailoring composition, porosity, and morphology, which makes them highly useful for Nb_2_O_5_− and ZrO_2_‐based structures; nonetheless, these advantages are often counterbalanced by challenges related to crystallinity control, pore uniformity, reproducibility, and process‐dependent heterogeneity. Electrospinning is particularly attractive for generating fibrous and scaffold‐like constructs, especially in Ta_2_O_5_‐containing composite systems designed for tissue engineering, yet such architectures may still require further optimization with respect to phase distribution, long‐term structural consistency, and interface homogeneity. In contrast, ALD offers unmatched conformality and nanoscale thickness control, which is especially valuable for Ta_2_O_5_ and HfO_2_ coatings on complex biomedical substrates, although these advantages are frequently achieved at the expense of increased process complexity, lower throughput, and reduced scalability. Accordingly, no single synthesis route can be considered universally optimal across all VMOs; rather, the most appropriate method depends on the target biomedical function, the oxide‐specific property profile, and the acceptable compromise between structural precision, manufacturing practicality, and translational relevance [55, 68, 69, 70, 71, 72, 73, 74, 75, 76].

## 3 Nanostructure Types and Synthesis Methods

The controlled fabrication of VMO nanostructures is fundamental to harnessing their unique properties for biomedical applications. The choice of synthesis method directly dictates critical morphological features, such as porosity, surface area, crystallinity, and topography, which in turn govern biological interactions, including protein adsorption, cell adhesion, and therapeutic efficacy. This section provides a comparative overview of the principal techniques employed to engineer these nanostructures. We examine five cornerstone fabrication strategies: electrochemical anodization for producing ordered nanotube arrays; the versatile sol–gel process for creating tailored nanoparticles and thin films; hydrothermal/solvothermal methods for crystallizing quantum dots and hierarchical structures; electrospinning for integrating oxides into polymeric nanofibrous scaffolds; and ALD for applying ultrathin, conformal coatings. Each method offers distinct advantages in controlling nanoscale architecture, directly linking synthetic parameters to the resulting physicochemical and biological performance outlined in subsequent sections.

### 3.1 Anodization

Anodization is a widely used nanofabrication technique that creates porous, well‐structured oxide layers on metals. It involves applying a voltage between an electrode and a counter electrode, submerged in a liquid electrolyte, thereby triggering electrochemical reactions that generate the oxide. The properties of this oxide, including its growth and structure, are affected by factors such as the applied voltage, the type of electrolyte, whether aqueous or organic, and the presence of etching agents, along with temperature and processing time [65].

A study by Khudhair et al. (highlights the significance of TiO_2_ nanotubes in medical applications, emphasizing their high corrosion resistance, biocompatibility, and ability to promote cell growth and mineralization [66]. These TiO_2_ nanotubes were fabricated by anodizing titanium and its alloys in fluoride‐based electrolytes, with their dimensions controlled by parameters such as voltage and electrolyte composition. The morphology of the nanotubes influenced their biocompatibility and electrical properties, which are crucial for applications such as cell adhesion, brain mapping, and in vivo studies. As a valve metal, titanium spontaneously forms a compact oxide layer upon exposure to oxygen. This oxide film protects the metal and provides high corrosion resistance. In that study, using titanium as the anode in an electrochemical anodization cell with a fluoride‐based electrolyte, a well‐organized TiO_2_ nanotube structure was successfully fabricated. The development of TiO_2_ nanotubes resulted from the competition between the growth and dissolution processes of the titanium oxide layer. The presence of fluoride ions in the electrolyte played a key role in this process. Moreover, the morphology of TiO_2_ nanotubes, including parameters such as diameter, length, and wall thickness, could be precisely controlled by adjusting anodization conditions, including voltage, electrolyte composition, pH, and duration.

### 3.2 Sol–Gel

The sol–gel process is a straightforward wet‐chemical technique used to produce materials such as oxides and nanomaterials, including nanoparticles, nanofibers, and nanoporous structures. It involves transforming selected monomers into a colloidal solution (sol), which then self‐assembles to form a gel. This coherent network can be deposited onto various substrates via methods such as spin or dip coating. This process offers advantages such as low‐temperature operation, control over composition and purity, and cost‐effectiveness. Key steps include hydrolysis, condensation, gelation, aging, drying, and densification. Although simple, it has limitations, such as weak bonding and difficulties controlling porosity and reaction rates. Its versatility and control make it popular for creating materials with excellent electrochemical performance and tailored structures for diverse applications. In a recent study [67], hierarchically porous niobium (V) oxide monoliths were synthesized through a sol–gel technique combined with phase separation and subsequent heat treatment. The study closely analyzed the pore architecture and crystal structures. Macropores were fine‐tuned by adjusting the initial composition to control phase separation, while mesopore structures were modified through air heat treatment to induce crystallization of niobium(V) oxide. Heating the dried gel at 550°C yielded a well‐structured macro‐ and mesoporous material with a surface area of 54 m^2^/g. Increasing the heat‐treatment temperature led to larger mesopores and a reduction in surface area, attributable to crystal growth.

Another study revealed that the biological effectiveness of Nb_2_O_5_ nanomaterials is largely influenced by particle size, shape, and surface features [68]. Smaller, well‐dispersed nanoparticles with high surface area tend to enhance antimicrobial activity, particularly against Gram‐negative bacteria such as *E. coli*, by disrupting cell membranes or generating ROS. The morphology, including crystalline phases and surface roughness, also played a role in determining how these particles interact with microbes. Additionally, the way particles agglomerate and their distribution within the material affected their consistency and effectiveness. Overall, the study revealed that optimizing these physical and chemical properties can significantly improve the antibacterial and ROS‐modulating capabilities of Nb_2_O_5_ nanomaterials, making them promising for various biomedical and environmental uses.

### 3.3 Hydrothermal and Solvothermal Methods

Hydrothermal and solvothermal methods are simple techniques for making metal oxide crystals. They involve dissolving a metal salt in a liquid and then heating the solution for a specified period. A recent study by Pratap et al. [69] presents an eco‐friendly hydrothermal synthesis of fluorescent L‐cysteine‐capped zirconium oxide quantum dots (L‐Cys‐ZrO_2_ QDs) [70]. The quantum dots, averaging 5.7 nm in size, exhibit characteristic UV–vis peaks at 320, 265, and 245 nm, with photoluminescence emission ranging from 360 to 500 nm and a quantum yield of approximately 3.6%. Surface functional groups such as –CO, –NH_2_, and –SO_4_^2−^ were confirmed via FT‐IR and XPS analyses. The QDs demonstrated stability in aqueous solutions, as evidenced by a zeta potential of approximately −26.3 mV, indicating good water solubility and stable photoluminescence. In vitro bioimaging experiments using HeLa cells showed successful cellular uptake and strong fluorescence under green laser excitation, confirming their potential as bioimaging labels. Cytotoxicity assays revealed low toxicity at 200 nM. The presence of elements like C and N, along with various functional groups, suggests effective surface passivation, making these QDs promising for biomedical applications. These structural features of ZrO_2_ QDs enhanced water stability, reduced agglomeration, and provided effective surface passivation, resulting in low cytotoxicity and excellent cell biocompatibility. The small size and surface chemistry also facilitate cellular uptake and strong photoluminescent labeling, making ZrO_2_ QDs ideal for bioimaging applications. The study's findings highlighted the potential of functionalized L‐Cys‐ZrO_2_ QDs as biocompatible, stable, and water‐soluble agents suitable for in vivo imaging and other biomedical applications.

### 3.4 Electrospinning

In a study, polycaprolactone (PCL) nanofibers with nanotantalum oxide (Ta_2_O_5_) were fabricated through electrospinning [53]. For electrospinning, a solution was prepared by dissolving PCL and Ta_2_O_5_ in trifluoroethanol at a 10% concentration, and stirred until homogeneous. This solution was loaded into a 10 mL syringe and extruded through a stainless‐steel needle. The electrospinning conditions included a flow rate of 1 mL/h with a syringe pump, an applied voltage of 13 kV, and a 15 cm distance between the needle tip and the collector. The resulting nanofibrous membranes were dried under vacuum for more than 48 h to remove residual solvent. These scaffolds were subsequently immersed in simulated body fluid (SBF) for 1–7 days to promote mineralization. The structural characteristics of Ta_2_O_5_, especially its surface hydroxyl groups and resulting scaffold morphology, played a vital role in its biological interactions. The surface hydroxyls distributed throughout the nanoparticle influence surface chemistry and hydration, both of which are key to bioactivity. These groups facilitated mineralization by attracting calcium ions and promoting hydroxyapatite formation, essential for bone tissue engineering. Additionally, they catalyzed polymerization reactions that modify scaffold properties, thereby enhancing cellular responses and degradation rates. As the Ta_2_O_5_ content increased, the scaffold's porosity and fiber morphology improved, supporting better cell infiltration and attachment. The study concluded that the presence, distribution, and type of surface hydroxyl groups, along with the scaffold's morphology, are crucial for optimizing Ta_2_O_5_'s bioactivity, mineralization potential, and biocompatibility.

### 3.5 Atomic Layer Deposition (ALD)

ALD is a precise technique for depositing ultrathin, uniform coatings at the atomic level. It operates via sequential, self‐limiting gas‐phase reactions that enable controlled film growth, typically one atomic layer per cycle. This method ensures high conformity and smoothness, even on complex or high‐aspect‐ratio surfaces, making it suitable for a wide range of materials, including oxides, nitrides, and metals. ALD's ability to coat various geometries on temperature‐sensitive substrates has made it essential in fields such as microelectronics, nanotechnology, and biomedical devices [71].

A recent study by Taratuta et al. [72] reported the surface modification of NiTi alloy occluders by applying a tantalum oxide (Ta_2_O_5_) coating via ALD to electrochemically polished surfaces. They performed physicochemical tests and in vitro biological evaluations, including cytotoxicity, cell proliferation, thrombogenicity, and proinflammatory cytokine assays. The study also assessed whether the coating improved fluoroscopic visibility. Results showed that the Ta_2_O_5_ coating enhanced the material's properties, notably decreasing cytotoxicity, thrombogenicity, and cytokine release, while encouraging cell proliferation. It significantly increased corrosion resistance, as evidenced by a fourfold increase in polarization resistance in undeformed samples and a twofold increase in samples deformed by 50%. Biocompatibility tests revealed lower cytokine levels and improved cell organization and density in coated samples that did not activate platelets. Although the coating did not substantially improve fluoroscopic visibility—likely due to its thinness on the wire—it overall improved the durability, corrosion resistance, and biocompatibility of NiTi occluders without affecting their pseudo‐elastic properties.

## 4 Physicochemical and Biological Characteristics

VMOs (especially titanium dioxide, zirconium dioxide, niobium pentoxide, and tantalum pentoxide) are among the most crucial semiconductor oxide classes used in the biomedical field. These oxides are usually designed as thin films with a thickness of 10–1000 nm, nanotubes with a diameter of 20–100 nm, or nanoparticles with a size of 200–500 nm, and the surface roughness is reported in the range of 1–50 nm in most studies [11, 13, 45]. For example, by preparing Nb_2_O_5_ thin films by the hydrothermal method, spherical nanoparticle morphology with a diameter of 200–500 nm and an average surface roughness of approximately 1.9 nm were obtained [15]. This size range directly affects the biological response at the implant‐tissue interface, as it provides the 10–100 nm scale topography, which is considered critical for protein adsorption and cell adhesion [12, 14, 73]. In the same study, it was reported that the Nb_2_O_5_ coating reduced the corrosion current density from 1.9 to 1.2 × 10^−2 ^ μA/cm^2^, providing approximately two orders of magnitude higher corrosion resistance. This suggests that long‐term stability in physiological environments is significantly improved [74, 75, 76].

The bandgap and semiconductor properties of these nanostructures are decisive for both electrochemical stability and ROS formation. The bandgap for TiO_2_ in the anatase phase is approximately 3.2 eV, while it is in the range of 5.0–5.5 eV for ZrO_2_, 3.1–3.4 eV for Nb_2_O_5_, and approximately 4.0–4.4 eV for Ta_2_O_5_ [47, 77, 78, 79, 80, 81, 82]. These values allow narrower bandgap oxides such as TiO_2_ and Nb_2_O_5_ to be photocatalytically activated under ultraviolet and limited visible light, while wider bandgap oxides such as ZrO_2_ and Ta_2_O_5_ behave as more inert, insulating, and mechanical barriers with high‐dielectric constants [13, 83, 84, 85] For example, Ta_2_O_5_'s bandgap of approximately 4.0 eV and the reported dielectric strength of ∼7 MV/cm contributed to both high electrical resistivity and the formation of a stable oxide layer on the implant surface [80, 86, 87]. When mechanical and electrical properties are evaluated together with pore size and porosity, porous tantalum skeletons in particular have been reported to show the best osteogenesis and osseointegration performance with a pore size of 400–600 µm and a porosity of approximately 75% [88, 89]. This pore spacing is compatible with the 300–700 µm range, which is necessary for new bone tissue to progress both to the surface and into the skeleton [90]. At the same time, it is emphasized that the compressive strength of such porous Ta structures can be adjusted to be close to the trabecular bone (e.g., 10–50 MPa range), thereby reducing the “stress shielding” effect [89, 91].

Surface chemistry and wettability are also critical parameters that determine the biological response. In a study comparing TiO_2_, ZrO_2_, Nb_2_O_5_, and Ta_2_O_5_ coatings produced by magnetron sputtering, it was shown that all coatings maintained cell viability at or above the control group level, but Nb_2_O_5_ and Ta_2_O_5_ coatings significantly increased mesenchymal stem cell (MSC) count and proliferation [11, 45, 92]. In the same type of metal oxide coatings, the water contact angles generally fall into the range of 60°–80° and the free surface energy increases to the order of 40–60  mJ/m^2^. This has been understood to facilitate the adsorption of serum proteins (e.g., albumin, fibrinogen) and integrin‐mediated cell adhesion [45, 93, 94, 95]. Recent studies on ZrO_2_ coatings and zirconia implants show that proper surface topography and wettability improve initial bone‐implant contact and soft tissue integration [96, 97].

Corrosion and tribocorrosion behavior are crucial for the long‐term success of metal alloy implants. In tribocorrosion studies comparing Nb_2_O_5_, TiO_2_, and combination coatings, it has been shown that the corrosion current density is one to two orders lower than that of uncoated surfaces. For example, in one study, the corrosion current density for the Nb_2_O_5 _ +  TiO_2_ dual‐phase coating was determined to be 2.57 × 10^−6 ^ A/cm^2^, 9.33 × 10^−7 ^ A/cm^2^ for TiO_2_ alone, and 2.21 × 10^−5 ^ A/cm^2^ for Nb_2_O_5_ alone. It has been stated that Nb_2_O_5_ has a more noble (positive) corrosion potential, while TiO_2_ shows a more pronounced passivation zone [45, 74, 75]. Similarly, in studies with sol–gel Nb_2_O_5_ films on Ti–Nb alloys, it was reported that the corrosion current density decreased from 1.36 × 10^−4^ to 7.37 × 10^−5 ^ mA/cm^2^ in physiological salt solutions and the corrosion potential shifted to more positive values, emphasizing that the corrosion resistance increased approximately twice [76]. Nanocomposite and hybrid coatings containing Ta_2_O_5_ have also been reported to reduce corrosion current density by up to one to two orders, increase passive film stability, and improve tribocorrosion resistance on NiTi and other [86, 98].

In terms of biological properties, VMO nanostructures are generally associated with good biocompatibility, controlled ROS production, and antimicrobial action. A 2022 study detailed the adhesion, viability, and osteogenic differentiation parameters of MSCs seeded onto TiO_2_, ZrO_2_, Nb_2_O_5_, and Ta_2_O_5_ thin films. All coatings are biocompatible, but the number of cells and the proportion of proliferative cells are significantly higher in Nb_2_O_5_ and Ta_2_O_5_ coatings than in other oxides [18, 92]. It is emphasized that semiconductive oxides, especially those with a bandgap in the 2–4 eV window, are suitable for smart implants that “respond to the pathogenic microenvironment” in a way that can produce a positive effect on osteogenesis and angiogenesis by creating a low dose of ROS, and an antitumor or antibacterial effect at high doses [83, 84, 99, 100, 101].

The numerical effects of surface nanotopography on cell behavior are well documented, particularly with TiO_2_ nanotubes. In a study comparing TiO_2_ nanotubes with diameters of 20, 50, and 100 nm, the adhesion of both human MSCs and osteoblasts on nanotubes with diameters of 50–100 nm increased significantly compared to surfaces with a diameter of 20 nm. Alkaline phosphatase (ALP) activity and mineralization, which are osteogenic markers, have been similarly reported to be higher [14, 102]. Another study published in 2025 showed that hierarchical TiO_2_ nanotube arrays significantly increased cell density and osteogenic gene expression compared to classical flat TiO_2_ surfaces, and this effect was particularly pronounced for nanotubes with diameters of 70–100 nm and multicascade nano‐microtopographies [11, 13, 16]. TiO_2_ nanotubes modulate osteogenic differentiation through epigenetic mechanisms and mechanotransduction pathways; recent studies also support the notion that it affects cell fate through YAP/Piezo1 and long noncoding RNA‐mediated signaling pathways [12, 103]. On the other hand, it is reported that bone marrow stem cell viability is significantly reduced and osteogenic markers such as Runx2 (Runt‐associated transcription factor 2) are suppressed in long‐term exposure of 70–100 nm nano TiO_2_ particles at high concentrations (e.g. 100 μg/mL and above), emphasizing that particle size and dose are critical in terms of toxicity [78, 104, 105, 106].

## 5 Surface Functionalization Strategies

Three main strategies have come to the fore to improve biofunctionality on VMO surfaces over the past 5  years: (i) polymer or biopolymer coatings, (ii) biomolecule immobilization, and (iii) creation of hybrid structures by doping. Polymer interlayers tens of nanometers thick are added on top of the oxide layers obtained by methods such as physical vapor deposition, sol–gel, microarc oxidation, and anodization, fine‐tuning both protein adsorption and cell adhesion [93, 107, 108, 109]. In a 2025 review, it was shown that customized physical vapor deposition coatings for medical devices can improve mechanical properties and wear resistance, as well as reduce corrosion current density by orders of one to two and strengthen the osteogenic response by keeping the thin film thickness controlled in the range of 100–1000 nm [17, 93].

In polymer coating strategies, it has become common to bond hydrogel‐like biopolymers (e.g., chitosan, carboxymethyl chitosan (CMCh), gelatin, collagen‐like polypeptides) to VMO surfaces to form layers 50–500 nm thick [93, 94, 109]. By coating the TiO_2_ nanotube arrays with a polymer system containing carboxymethyl chitosan/alendronate/Sr^2+^, these hybrid surfaces have been shown to significantly increase both osteogenic activity and antibacterial efficacy [110]. In the same study, it was reported that the ALP activity of osteoblast‐like cells increased by approximately 1.5–2 times and the number of bacterial colonies decreased by the order of one log on the modified surface compared to the uncoated TiO_2_ nanotube surface [110]. The addition of chitosan or its derivatives to metal oxide surfaces both strengthens the electrostatic interaction with the cell membrane by adjusting the surface charge and hydrophobicity, and creates an antimicrobial barrier with the controlled release of silver, zinc, or copper ions [111, 112, 113, 114].

Biomolecule immobilization is used to locally present bone morphogenetic proteins, arginine‐glycine‐aspartate (RGD) motif peptides, and angiogenic factors, especially on porous tantalum and TiO_2_ nanotube surfaces. In studies in which RGD peptides were covalently bonded to porous tantalum skeletons, it was shown that in skeletons with a pore size of 400–600 μm (micrometers) and 75% porosity, new bone formation increased significantly both at the interface and inside the pores in the RGD‐modified groups, and mechanical bonding strength was considerably higher than in the unmodified group [89, 99]. Two reviews published in 2022 and 2025 highlight that the functionalization of tantalum and porous tantalum surfaces with calcium phosphate, collagen, peptides, and antibiotics increases osseointegration rate and implant‐bone interface strength, especially in the first 4–8  weeks, while also reducing the risk of infection [83, 91].

Doping and hybridization strategies are used both to adjust the bandgap and to control functions such as ROS generation, ion release, and electrical conductivity. It has been reported that in systems where Nb_2_O_5_ thin films are used in combination with WO_3_, the optical bandgap can be reduced from 3.75 to 3.10 eV by increasing the WO_3_ concentration, thus significantly improving the photocatalytic and electrochemical response under visible light [115]. Similarly, it has been shown that the bandgap of Ta_2_O_5_ can be lowered from about 4.0 to 2.4 eV by nitrogen doping, thereby developing functional coatings that can be activated by lower energy photons [86, 116]. Recently published semiconductive biomaterials articles discuss in detail that ZnO, TiO_2_, Nb_2_O_5_, and Ta_2_O_5_‐based heterojunctions obtained by such bandgap engineering can provide multifunctional responses such as controlled ROS production under light or electric field, antibacterial effect, and modulation of the tumor microenvironment [83, 84, 85, 99].

In studies combining ion doping with TiO_2_ nanotubes, it has been reported that both osteogenic and antibacterial effects can be increased simultaneously, especially when combined with strontium ions, carbon monoxide capture molecules, and drugs that regulate bone metabolism. TiO_2_ nanotube coatings modified by the combination of Sr^2+^ and CO have been shown to both significantly increase mineralization in osteogenic cells and strengthen antibacterial activity against Gram‐positive and Gram‐negative bacteria [117]. Multicomponent systems, such as TiO_2_‐Hap‐Ag or HAp‐binding silver/TiO_2_ hybrids, have also been shown to achieve a balanced performance between photocatalytic antibacterial activity, enamel remineralization, and biocompatibility [118, 119, 120, 121]. Nanotubes with diameters of 50–100 nm and modified layer thicknesses of several hundred nanometers are often reported on such surfaces, providing both sufficient volume for ion release and the necessary strength to maintain mechanical integrity [12, 14, 122, 123].

Finally, multicomponent hybrid systems combine VMOs with polymer matrices, bioceramics, and conductive phases. For example, semiconductive coatings and osteogenic drug‐releasing systems integrated into 3D‐printed calcium phosphate skeletons significantly increased mineralization and new bone volume compared to control groups; In some studies, reports of increases in bone volume in the range of 20%–40% at 4–8  weeks follow‐up are reported depending on drug loading and release parameters [85, 99, 124, 125]. Reviews covering the period 2020–2025 reveal that corrosion current density can be reduced by one to two orders, surface hardness can be increased by 2–3 times, and osteogenic differentiation can be strengthened by maintaining cell viability by generally over 90% thanks to such hybrid structures [17, 77, 92, 109, 126].

VMO nanostructures (especially titanium dioxide, tantalum pentoxide, aluminum oxide, and Nb/Zr‐based oxides) possess a multistep interface chemistry coordinately controlled by missing metal centers (M^n+^) and hydroxyl‐rich surface groups (≡M—OH); this structure enables both ligand exchange and condensation‐based covalent functionalization [83, 84]. The mechanism proceeds primarily through activation of surface hydroxyl groups (≡M—OH  ⇌  ≡M—O^*−*^ + H^+^) followed by nucleophilic substitution/dehydration steps; During this process, stable M—O—Si, M—O—C, and M—O—P bridges are formed as a result of reactions such as silanization (≡M—OH + R—Si(OR′)_3 _ →  ≡M—O—Si—R + 3R′OH), carboxylate coordination (≡M—OH + HOOC—R  →  ≡M—OOC—R  +  H_2_O), and phosphate bonding (≡M—OH + R—PO(OH)_2 _ →  ≡M—O—PO(OH)—R  +  H_2_O). However, these bonds are not purely covalent and exhibit a distinct ionic‐covalent hybrid character due to the high oxidative power and Lewis acidity of valve metals; this is directly related to interfacial dipole formation, charge redistribution, and electron‐transfer processes. Redox processes (Ti^4+ ^ +  e^*−*^ →  Ti^3+^, O_2 _ +  e^*−* ^  →  O_2_•^*−*^) developing particularly through oxygen vacancies (V_0_••) and low‐coordination metal centers enhance surface reactivity, thereby strengthening binding energy and kinetics [83, 85]. Kinetically, the system is characterized by Langmuir–Hinshelwood or Elovich models, depending on surface saturation and active‐site density; pseudo‐first‐order behavior is observed in the initial phase, while chemisorption‐controlled pseudo‐second‐order kinetics prevail in later phases, and the rate constants are determined by pH, surface hydration, precursor concentration, and steric accessibility. In this context, functionalization density (Γ) is a key determinant not only of reaction kinetics but also of the biological response at the nanobiological interface: at low Γ values, protein adsorption and cell adhesion remain weak due to limited ligand delivery, while in the mid‐range, integrin‐mediated signaling reaches its maximum thanks to optimal ligand spacing and receptor access; conversely, at high Γ values, biological activity decreases due to steric hindrance, surface charge repulsion, and biomolecular conformational constraint. This nonlinear relationship is often described by bell‐curve behavior in the form B  ∝  Γ·exp(−*α*Γ), indicating a narrow window of optimal functionalization [86]. Furthermore, modification of the nanostructure's specific surface area, mesoporosity, and band structure through doping enhances both chemical binding efficiency and biological interactions by increasing the density of active sites and the kinetics of electron transfer [87]. In conclusion, the relationship among the interface‐binding mechanism, reaction kinetics, and functionalization density in valve metal–oxide nanostructures reveals a quantitative, finely balanced structure‐function‐bioresponse correlation determined by surface hydroxylation, defect chemistry, ligand architecture, and the energy landscape.

## 6 Biomedical Applications

**FIGURE 3:**
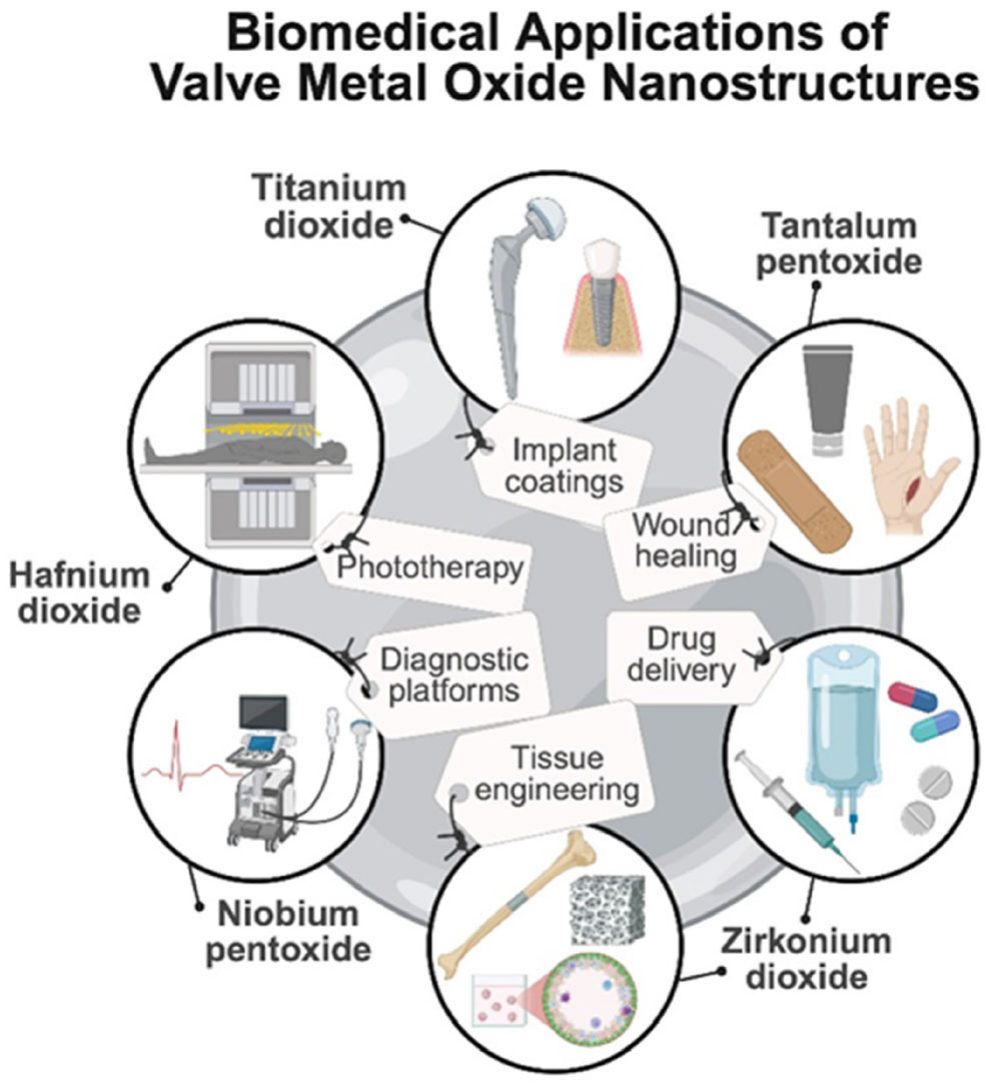
Biomedical applications of valve metal oxide nanoparticles: showing their popularity in modern biomedicine. Their unique nanoscale features enable targeted interaction with biological environments, opening pathways for diverse applications such as implant coatings that enhance tissue integration, antibacterial surfaces that inhibit biofilm formation, and nanocarriers for targeted drug release. Additionally, their optical and electrochemical properties position them as promising candidates for biosensing and phototherapeutic strategies.

VMO nanostructures have become increasingly popular in modern biomedicine, owing to their biocompatibility, tunable surface chemistry, and structural stability. Their unique nanoscale features enable targeted interaction with biological environments, opening pathways for diverse applications such as implant coatings that enhance tissue integration, antibacterial surfaces that inhibit biofilm formation, and nanocarriers for targeted drug release. Additionally, their optical and electrochemical properties position them as promising candidates for biosensing and phototherapeutic strategies. The following sections provide an overview of these application areas, summarizing recent advances and highlighting the functional potential of these nanomaterials in clinical and translational contexts. Some biomedical applications of VMO nanoparticles are shown in Figure 3.

### 6.1 Implant Coatings and Surface Engineering

Despite many advances in implant design and materials, insufficient or delayed osseointegration remains a persistent challenge in clinical implantology. To address this limitation and promote bone formation following implant placement, a variety of surface modification and coating strategies have been developed [88]. Surface engineering of orthopedic and dental implants using VMO coatings such as TiO_2_, Ta_2_O_5_, and Nb_2_O_5_ represents a promising strategy to enhance osteointegration, corrosion resistance, and long‐term biocompatibility. These oxides exhibit excellent chemical stability, favorable mechanical properties, and bioinert‐to‐bioactive surface behavior, making them particularly suitable for permanent implant applications. Nanostructured coatings further improve biological performance by modulating cell with surface interactions and promoting integration of implant into tissue [18, 127].

Titanium dioxide‐based coatings remain among the most widely applied surface modifications for metallic implants. Nanotubular and nanostructured TiO_2_ coatings have been shown to significantly enhance osseointegration by promoting osteoblast adhesion, differentiation and mineralization: in vivo and in vitro studies demonstrate that TiO_2_ nanotubes improve bone–implant contact and support osteogenic differentiation, highlighting their clinical relevance in orthopedic implant surfaces [127]. An in vivo study showed that implants coated with TiO_2_ nanotubes led to a significally increased gene expression of osteogenesis‐related genes like ALP in bone samples around the coated implant and an increased contact of bone and implant, which was attributed to the structure of the nanotubes [128]. An in vitro experiement showed an increased adhesion of osteoblasts on titanium, when coated with TiO_2_ nanotubes by a remarkable 300%–400% compared to the plain Titanium surface [129]. Similar results were also confirmed in vivo by an increased bone‐to‐implant attachment in rabbits [130]. Coating TiO_2_ nanotubes with additional tantalum was shown to lead to an even greater ALP activity (indicating osteoblast activity) and a 30% increased rate of matrix mineralization and bone nodule formation compared to only TiO_2_ nanotubes in human osteoblast cells [131].

Not only TiO_2_ nanostructures, but Ta_2_O_5_ nanotube array films were also shown to increase the corrosion resistance and hydrophilicity compared to plain tantalum, leading to increased protein adsorption. This study furthermore suggested that Ta_2_O_5_ nanotube films increase the biocompatibility of plain tantalum by increasing osteoblast differentiation of bone MSCs from rabbits [34]. Another in vitro study confirmed increased osteoblast activity on Ta_2_O_5_ coated scaffolds by an increased proliferation, an enhanced osteoblastic gene expression (ALP, RUNX2) and greater mineralization, indicating for increased osteoblast activity [132]. Comprehensive evaluations of niobium‐based biomaterials additionally indicate excellent cytocompatibility, enhanced osteogenic responses, and long‐term stability, supporting their potential use in next‐generation orthopedic implants [95].

### 6.2 Antimicrobial Surfaces and Wound Healing

Coating surfaces (i.e. implants) with nanostructures not only leads to an enhanced integration of coated implants into the tissue and bones, displayed by an increased osteoblast adhesion, but can also exhibit antimicrobial properties: VMO‐based antimicrobial surfaces, again, especially those involving TiO_2_ nanostructures and nanocoatings, have been investigated due to their light‐activated antibacterial functionality. Several studies report that nanostructured TiO_2_ coatings effectively reduce bacterial adhesion and proliferation on biomedical surfaces while maintaining compatibility with mammalian cells [133, 134, 135].

The predominant antibacterial mechanism of TiO_2_‐based coatings is due to photocatalytic generation of ROS, like hydroxyl radicals and superoxide anions [135, 136, 137]. Photocatalytic generation of ROS refers to the light‐induced excitation of semiconductor materials (such as VMO nanostructures), leading to the formation of electron hole pairs (e^−^/h^+^) that react with molecular oxygen and water to produce ROS [101, 102]. These ROS can induce oxidative damage to bacterial membranes, proteins, and nucleic acids, leading to their inactivation or killing [96]. Because ROS are generated locally at the coated implant surface, cytotoxic off‐target effects toward surrounding mammalian cells are expected to remain limited.

The most used photocatalytic agent is TiO_2_ [137, 138]. The generation of ROS is independent of any antibiotic pathways, making nanoparticle coatings a promising tool in the context of a global rise in antimicrobial resistance [139].

Through the specific design of the applied material, both ROS generation as well as their biological effects can be precisely controlled. For instance, doping strategies can enhance the overall photocatalytic capacity and consequently increase antibacterial activity. For example, doping TiO_2_ nanomaterials with silver (Ag), copper (Cu), or cerium (Ce) has been shown to enhance ROS production and improve antibacterial efficacy against *Streptococcus mutans* [105].

Importantly, TiO_2_‐mediated antibacterial activity is not only determined by the total amount of ROS generated, but also by the type of ROS produced. The main photocatalytic ROS generated at TiO_2_ surfaces include hydroxyl radicals (•OH), superoxide radicals (O_2_•^−^), singlet oxygen (^1^O_2_), and hydrogen peroxide (H_2_O_2_) [96]. However, the exact type of ROS generated plays an important part in terms of cytotoxicity and off‐target effects and can also be achieved by surface modification of plain TiO_2_ nanomaterials. For example Ma et al. demonstrated that modulation of the TiO_2_ crystal phase composition, particularly the anatase/rutile ratio, can alter ROS selectivity and promote preferential O_2_•^−^ generation at the nanoparticle surface [106]. In contrast, crystal facet engineering has been associated with enhanced •OH formation. Specifically, highly reactive anatase TiO_2_ {001} facets favor hole‐driven surface oxidation reactions, thereby promoting the generation of •OH radicals [107]. These findings indicate that targeted control over TiO_2_ crystal structure and surface facets may enable selective tuning of photocatalytic ROS production and its associated biological effects. Another mechanism to selectively generate O_2_•^−^ radicals is trough the incorporation of surface defects trough oxygen vacancies. By this, trapping sites for charge carriers can be generated. This promotes the reduction of O_2_ to O_2_•^−^ [108]. To specifically generate singlet oxygen species ^1^O_2_, Saito et al. showed that TiO_2_ surfaces can be modified with gold nanoparticles (AuNPs). Upon visible‐light irradiation, electrons are transferred from the Au nanoparticles to TiO_2_, leading to the formation of superoxide radicals O_2_•^−^. These O_2_•^−^ radicals can subsequently be oxidized by positive holes remaining in the Au nanoparticles, resulting in the formation of ^1^O_2_ [109].

The importance of fine tuning the type of ROS generated lies in the toxicity and off‐target effects. Hydroxyl radicals (•OH) exhibit extremely high reactivity and can induce rapid and largely nonselective oxidative damage to lipids, proteins, and nucleic acids [140]. While this may enhance antibacterial efficacy, excessive •OH generation may also increase cytotoxicity toward mammalian cells, as this highly reactive molecule can react with any type of cell [140]. In contrast, superoxide radicals (O_2_•^−^) exhibit comparatively lower reactivity and primarily contribute indirectly through ROS cascade formation and secondary oxidant generation [140], potentially reducing immediate off‐target cytotoxicity compared to •OH radicals.

Nevertheless, several studies have already indicated that ROS generated by TiO_2_ are not substantially harmful to mammalian cells. For instance, Nica et al. demonstrated pronounced antibacterial and antifungal activity of TiO_2_‐based nanomaterials against several clinically relevant microbial species, including *Candida albicans*, *Staphylococcus aureus*, *Enterococcus faecalis*, *Escherichia coli*, and *Pseudomonas aeruginosa*, while simultaneously showing good biocompatibility toward mammalian dermal and lung fibroblasts under the investigated conditions [110].

Combining TiO_2_ coatings with noble metals, transition metals, or antibiotics further enhance antibacterial efficacy through synergistic mechanisms, including also contact‐based bacterial damage trough antibiotics and increased photocatalytic efficiency trough metals [137]. Such composite nanocoatings demonstrate antibacterial performance against both Gram‐positive and Gram‐negative bacteria and are therefore attractive for infection‐resistant implant surfaces, medical devices, as well as wound‐contact materials [133].

In wound‐healing‐related applications, TiO_2_‐based antimicrobial nanocoatings offer the advantage of localized and externally controllable antibacterial activation through light exposure, enabling efficient surface sterilization while minimizing systemic toxicity [137, 138]. Light‐activated disinfection describes the antimicrobial strategy in which photocatalytic surfaces are irradiated with UV or visible light to induce rapid and broad‐spectrum antimicrobial activity. This approach has demonstrated efficacy against Gram‐positive and Gram‐negative bacteria and has been shown to inhibit microbial adhesion and biofilm formation on medical implants and wound dressings [133, 137, 138].

Overall, VMO‐based nanocoatings, particularly those based on TiO_2_, represent a multifunctional surface engineering strategy that simultaneously promotes implant integration and provides light‐activated, antibiotic‐independent antimicrobial protection, making them highly promising for infection‐resistant implants, medical devices, and wound‐healing applications.

### 6.3 Nanocarriers and Drug Delivery

Nanocarriers hold a lot of potential for targeted treatment of many diseases as they can be engineered to target specific cell‐surface receptors and are internalized into cells very succesfully [141]. Nanostructured VMOs, including TiO_2_, Ta_2_O_5_, Nb_2_O_5_, ZrO_2_, and HfO_2_, have emerged as promising platforms for controlled drug delivery owing to their chemical stability, biocompatibility, and adaptable nanoscale architectures. Mesoporous and tubular nanostructures provide high specific surface areas and well‐defined pore networks, enabling efficient drug loading and tunable release kinetics. Among these materials, TiO_2_ has been most extensively investigated, demonstrating broad applicability for the delivery of chemotherapeutics, antibiotics, and bioactive molecules [123, 142].

Especially nanostructured TiO_2_ can be used as nanocarriers for therapeutic agents such as genes, drugs, or antigens [123]. For example, one study reported the successful packaging of doxorubicin into TiO_2_ nanotubes and a subsequent release, reporting them as promising drug‐carriers [143]. Tubular TiO_2_ nanostructures produced by anodization offer vertically aligned nanochannels with controllable diameter and length, which directly influence drug encapsulation efficiency and release behavior. These systems have again been shown to successfully load anticancer agents such as doxorubicin and support sustained drug release, with loading mechanisms influenced by surface interactions and nanotube geometry [143]. Beyond passive carrying, TiO_2_ nanotubes and nanocylinders can be internalized by cells, facilitating intracellular drug delivery and improving therapeutic efficacy [141].

Though TiO_2_ are the most investigated nanostructure, other VMOs have gained increasing attention as drug delivery platforms as well. Mesoporous tantalum oxide nanoparticles have been developed for high‐capacity drug loading and have shown enhanced therapeutic outcomes in synergistic chemoradiotherapy while reducing systemic toxicity [144]. Similarly, Ta_2_O_5_‐polymer hybrid nanostructures enable controlled drug release through pH‐sensitive mechanisms, highlighting their potential for targeted therapy [145].

Though less explored for drug delivery, niobium pentoxide nanostructures have tunable nanostructural properties and surface reactivity, making them promising candidates for biomedical applications, potentially drug delivery [146].

Zirconium‐based nanostructures further expand the toolbox of VMO nanocarriers. Hollow and mesoporous zirconia nanospheres have demonstrated dual pH‐responsive drug release behavior, enabling controlled payload liberation under acidic conditions, as found in the tumor microenvironment [147]. In addition, zirconium‐based frameworks and hybrid nanostructures have been explored for targeted drug delivery applications, showing efficient loading and regulated release profiles [148].

Stimuli‐responsive drug delivery represents a major advancement in VMO‐based nanocarrier design. pH‐sensitive systems exploit pathological microenvironments, such as the acidic tumor milieu, to trigger drug release from mesoporous or hollow oxide architectures [145, 147]. Photoresponsive platforms, particularly based on TiO_2_ nanotubes, enable externally controlled drug release through ultraviolet, visible, or near‐infrared light irradiation, providing precise spatial and temporal regulation while minimizing off‐target effects [149]. Furthermore, drug release can be controlled via other mechanisms like temperature, ultrasound or radiofrequency [150].

Emerging systems based on hafnium oxide nanostructures further illustrate the broad spectrum of VMOs in smart drug delivery. HfO_2_‐based nanotherapeutics have been engineered to combine pH sensitivity with enhanced radiotherapeutic performance through HfO_2_, supporting synergistic treatment strategies and enabling deep‐tumor penetration [151].

Overall, nanostructured VMOs constitute a highly versatile class of drug delivery platforms that combine high loading capacity, tunable and stimuli‐responsive release, and favorable biocompatibility, enabling localized, targeted, and multifunctional therapeutic strategies for next‐generation nanomedicine.

### 6.4 Biosensors and Diagnostic Platforms

Biosensors and diagnostic platforms are analytical systems that combine so‐called biological recognition elements like proteins, enzymes, nucleic acids, receptors, antibodies, or whole cells, with a physicochemical transducer to detect specific biomolecules. The interaction between the target analyte and the functionalized surface is converted into a measurable electrical or optical signal, enabling sensitive, selective, and often real‐time detection relevant to disease diagnosis, monitoring, and therapeutic decision‐making [152, 153].

VMO‐based nanomaterials have emerged as functional components in biosensors and diagnostic platforms due to their semiconducting and dielectric properties, which enable efficient transduction of biomolecular recognition events (from proteins, enzymes, nucleic acids, receptors, antibodies, or whole cells) into measurable electrical or optical signals [152, 153]. The semiconducting nature of these nanomaterials enables changes in charge distribution, conductivity, or interfacial impedance upon biomolecule binding, allowing highly sensitive detection of proteins, nucleic acids, and small‐molecule biomarkers and other biomolecules [154, 155].

Electrochemical biosensors exploit changes in current, potential, or impedance arising from biomolecular interactions at the electrode interface. Nanostructured metal oxides significantly enhance these signals by increasing electroactive surface area, facilitating charge transfer, and enabling high‐density immobilization of biorecognition elements such as enzymes, antibodies, or nucleic acids [152, 155].

In electrochemical biosensors, metal oxide nanostructures such as TiO_2_, ZnO, and related semiconducting oxides are widely used to enhance sensitivity by improving electron transfer kinetics, increasing electroactive surface area, and facilitating stable immobilization of biorecognition elements [152, 155]. The semiconducting behavior of these nanomaterials allows biomolecule binding events to be detected through changes in electrical properties such as surface charge, conductivity, or impedance [155].

Optical biosensors rely on changes in optical properties such as absorbance, fluorescence, refractive index, or photoluminescence upon biomolecular binding. VMOs are particularly well suited for optical sensing due to their high refractive indices, dielectric constants, and tunable band structures, which enable strong light–matter interactions at the nanoscale [153, 156].

### 6.5 Phototherapy and Imaging

Due to their photoactive and semiconducting properties, their high photostability as well as compatibility with multifunctional nanocomposite designs, VMOs have gained significant attention in phototherapy and biomedical imaging. In cancer therapy, these materials are increasingly explored in PDT and photothermal therapy (PTT), where light‐induced processes are exploited to induce localized cytotoxic effects while minimizing damage to healthy tissues. Recent advances emphasize the integration of VMOs into composite nanomaterials to enhance therapeutic efficacy, targeting specificity, and imaging performance [157].

PDT relies on light‐triggered excitation of photosensitizers, which results in the generation of ROS, particularly singlet oxygen, which induces oxidative damage and can lead to (cancer) cell death [158, 159]. Metal oxide‐based nanomaterials can act as photosensitizers themselves or as carriers that enhance photosensitizer stability, cellular uptake, and light responsiveness [160]. Semiconducting oxides such as TiO_2_ possess strong photocatalytic activity and can generate ROS upon photoexcitation, making them suitable candidates for PDT‐related applications [161]. Metal oxide nanostructures, such as titanium dioxide TiO_2_, can furthermore be engineered to act as photosensitizers or carriers, improving singlet oxygen generation and tumor selectivity. Additionally, nanomaterials can be functionalized with targeting to enhance tumor accumulation and reduce off‐target effects [158, 159].

PTT, on the other hand, exploits the efficient conversion of absorbed (mostly near infrared) light into heat, leading to localized hyperthermia and (tumor) cell death [133]. Semiconductor nanostructures, to which VMO nanostructures belong, are mechanistically well suited for PTT [134].

Nanostructured inorganic semiconductors with strong NIR absorption are particularly effective photothermal agents. While VMOs may exhibit limited intrinsic NIR absorption, their functional integration into composite nanomaterials enables enhanced photothermal performance through synergistic interactions with plasmonic, carbon‐based, or other semiconductor components [135, 136]. Such hybrid architectures allow controlled heat generation under NIR irradiation, offering spatially confined tumor treatment and reduced systemic toxicity [134].

Recent studies highlight the functional integration of metal‐based nanocomplexes that combine PTT with bioimaging capabilities in one single platform. Such systems use the chemical stability and surface composition of inorganic oxides to enable targeted accumulation at target cells, imaging signal enhancement, and synergistic therapeutic effects [137]. Composite nanomaterials incorporating VMOs further enable multifunctionality, allowing precise tumor targeting through surface functionalization while supporting combined PDT/PTT or imaging‐guided therapy [162].

The convergence of phototherapy and imaging represents a key advancement in cancer theranostics. VMO‐based nanocomposites can support multimodal imaging techniques, including photoacoustic, fluorescence, and optical contrast imaging, enabling real‐time monitoring of therapeutic delivery and treatment response [138]. In particular, inorganic nanostructures optimized for photoacoustic imaging combine strong optical absorption with efficient thermal expansion, facilitating high‐contrast, deep‐tissue imaging while simultaneously guiding PTT [138].

Overall, VMO‐based nanostructures and composite materials represent a powerful toolkit for phototherapy and biomedical imaging. Through rational nanostructure design and integration into multifunctional composites, these systems enable controlled ROS generation, efficient photothermal conversion, and enhanced imaging contrast. Continued developments in this field are expected to advance targeted cancer therapy and imaging‐guided treatment strategies, further solidifying the role of VMOs as versatile platforms for next‐generation phototheranostics.

### 6.6 Tissue Engineering

VMO nanostructures play an important role in tissue engineering by enabling precise control over cell–material interactions through nanoscale surface engineering. In particular, anodically formed TiO_2_ nanotubes and related nanostructures provide tunable nanotopographical signals that directly regulate cell adhesion, proliferation, and lineage‐specific differentiation, which are critical parameters for regenerative applications in bone and dental tissues [139].

**FIGURE 4:**
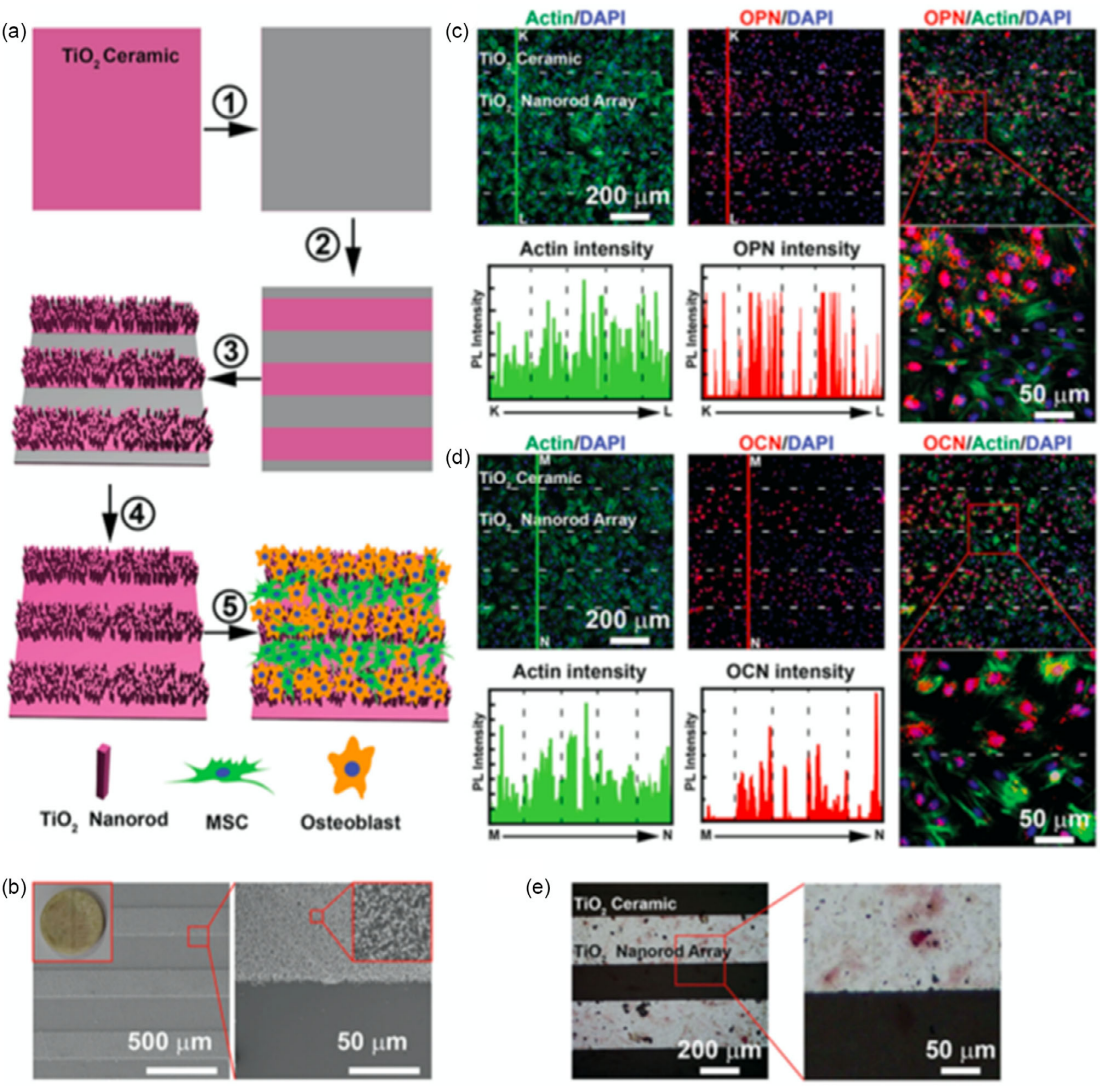
Location‐committed osteogenic differentiation of MSCs on a micropatterned TiO_2_ substrate. (a) Schematic of the fabrication process: (1) photomask spin‐coating, (2) photolithography, (3) hydrothermal growth of a TiO_2_ nanorod array, (4) photomask removal, and (5) Mesenchymal stem cells (MSCs) culturing. (b) Digital and SEM images of the fabricated patterned substrate. Immunofluorescence staining and relative expression level analysis of osteogenic markers (c) osteopontin (OPN) and (d) osteocalcin (OCN) after 14  days of culture. (e) Calcium nodules stained with Alizarin Red S. Adapted with permission [163]. Copyright 2019, Wiley‐VCH.

Since nanotopographical features influence cell adhesion, proliferation, and differentiation, nanotube‐based surface engineering provides a powerful strategy to optimize biomaterial–cell interactions. One engineering strategy is choosing the right nanotube diameter. The diameter of TiO_2_ nanotubes was shown to critically influence adhesion and attachment. Furthermore, the diameter influences osteogenic differentiation as shown by an enhanced osteogenic marker expression of osteoblast like cells, like RUNX2 and osteocalcin [141]. Not only diameter but also spacing, and hierarchical organization can influence initial cell adhesion by determing sites of attachment of cells, cytoskeletal organization, and integrin‐mediated signaling pathways [142]. Hierarchical TiO_2_ nanotube architectures combining micro‐ and nanoscale features have been shown to enhance osteoblast proliferation while simultaneously promoting osteogenic differentiation through mechanotransductive signaling mechanisms (Figure 4) [163].

Not only osteoblast‐like cells, but also MSCs are particularly sensitive to nanoscale surface features, and multiple studies report that TiO_2_ nanotube arrays can direct MSC fate without the need for exogenous biochemical factors [13, 164]. Again, hierarchically organized TiO_2_ nanotube arrays have been shown to enhance MSC adhesion, elongation, and later osteogenic differentiation by promoting cytoskeletal tension and nuclear deformation, which are key regulators of mechanosensitive gene expression [13].

The osteoinductive potential of VMO nanostructures can be further enhanced through surface functionalization strategies. Covalent immobilization of osteogenic growth factors, such as platelet‐derived growth factor‐BB (PDGF‐BB), onto clustered TiO_2_ nanotubular surfaces has been demonstrated to synergistically enhance bone marrow‐derived MSC differentiation and mineralization, combining topographical and biochemical cues in a single application [165].

In addition to titanium‐based systems, other VMOs such as tantalum oxide have emerged as promising candidates for bone tissue engineering. Nanolamellar tantalum interfaces exhibit excellent osteoblast adhesion and spreading behavior, attributed to their favorable surface energy and nanoscale roughness, further showing the broader relevance of VMO nanostructures beyond TiO_2_ [166].

In the context of dental tissue engineering, nanotopography‐mediated regulation of stem cell behavior is equally critical. Nanoscale surface features have been shown to direct the osteogenic and odontogenic differentiation of human dental pulp‐derived stem cells, highlighting the potential of VMO nanostructures for dental regeneration and implant integration [167].

Collectively, these findings demonstrate that VMO nanostructures serve as powerful surface‐engineering tools capable of enhancing cell adhesion, proliferation, and differentiation through precisely defined nanotopographical designs. Their demonstrated efficacy in bone and dental regeneration places them as key material platforms for next‐generation tissue‐engineered implants and regenerative therapies.

## 7 Toxicity and Biocompatibility

In order to translate VMO nanocoatings into clinical applications, understanding the toxicity of the applied nanoparticles and structures is essential.

Although this is an indended antibacterial mechanism, a central part responsible for metal oxide nanoparticle‐induced cytotoxicity is the generation of oxidative stress. Many metal‐based nanomaterials catalyze the formation of ROS, either intrinsically or following cellular internalization, leading to lipid peroxidation, protein oxidation, mitochondrial dysfunction, and DNA damage [168, 169]. However, cytotoxicity arising from nanomaterials is dependent on many factors, including, for instance, concentration and time. At low concentrations, certain oxide nanomaterials have only minimal effects, while higher doses can induce apoptosis, necrosis, or suppression of cell proliferation [170]. Furthermore, the material of the nanoparticles is essential for its cytotoxicity, with TiO_2_ NPs being the least and CuO being the most cytotoxic [171]. In order to assess cytotoxicity, the material and concentration of nanomaterials need to be carefully assessed. Apart from material and concentration considerations, toxicity needs to be assessed target‐cell‐specific. Mechanistic investigations demonstrate that different cell populations, such as hepatocytes and macrophage‐like Kupffer cells, exhibit divergent cell death pathways in response to metal‐based engineered nanomaterials, highlighting the relevance of target‐cell context in toxicity assessment [172].

Recent advances in toxicogenomics and nanotoxicomics have provided deeper insights into the molecular pathways induced by metal oxide nanoparticles. Global transcriptomic and epigenetic analyses reveal that valve metal oxid nanostructure exposure can alter genes involved in oxidative stress response, inflammation, cell cycle regulation, and DNA repair, even in the absence of obvious cytotoxicity [106, 173]. These findings highlight the importance of integrating molecular‐level investigations into biocompatibility assessments rather than relying only on conventional viability assays and cytotoxicity assessment.

Robust evaluation of metal oxide nanoparticle toxicity requires standardized and both in vitro and in vivo testing strategies. In vitro testing in diverse cell lines representing different tissues like respiratory, intestinal, dermal, hepatic, and immune cells enables identification of organ‐specific responses to nanostructures as well as toxic‐dose thresholds [154]. Integrated approaches combining oxidative stress biomarkers, redox‐related assays, and in vivo validation provide a more physiologically relevant assessment of nanomaterial safety and systemic effects [155].

In vivo and occupational exposure studies further highlight the relevance of long‐term and low‐dose effects. Biomonitoring of individuals exposed to metal oxide nanoparticles reveals associations with oxidative stress markers and epigenetic alterations, emphasizing the importance of chronic exposure assessment alongside acute toxicity testing [156]. Moreover, microbial and environmental studies demonstrate that metal oxide nanoparticles can exert harmful effects across biological systems, reinforcing the need for careful lifecycle and safety evaluation [157].

Valve metal (e.g., Ti, Nb, Ta, Zr) nanostructures are well established to form dense, self‐passivating oxide films characterized by high thermodynamic stability in aqueous and oxidizing environments, broad chemical inertness across a wide pH range, and strong interfacial adhesion to the metallic substrate. In particular, titanium‐based alloys spontaneously develop a native TiO_2_ passive layer with a thickness on the order of 2–10 nm, which effectively suppresses ionic release and constitutes the fundamental basis of their biocompatibility. Similarly, oxides such as TiO_2_, Ta_2_O_5_, and Nb_2_O_5_ exhibit high hardness, extremely low solubility, and excellent corrosion resistance. Consequently, the dissolution kinetics and ion release from VMO nanostructures are negligible under physiological conditions, rendering them largely inert toward biological media; measurable ion release typically occurs only upon mechanical or chemical disruption of the passive film [158]. Nanostructured VMOs function as highly effective barrier layers, significantly reducing corrosion currents and inhibiting ionic leaching. For instance, TiO_2_ nanotubular arrays and thin films have been shown to markedly decrease corrosion rates in SBFs [159], while Ta_2_O_5_ and Nb_2_O_5_ coatings provide robust protection even for highly reactive substrates such as magnesium alloys [160]. More broadly, VMO nanostructures serve as efficient nanofillers in polymer‐based coatings, where they contribute to the formation of dense, impermeable matrices that restrict the ingress of aggressive species. The associated corrosion protection mechanisms include physical barrier effects, surface passivation, rapid self‐repassivation, and improved coating adhesion. In addition to their protective role, these nanostructures impart significant biological advantages, including enhanced biocompatibility, reduced bacterial adhesion, and promotion of cellular proliferation and osteointegration. Notably, VMOs exhibit rapid repassivation following localized damage (e.g., abrasion or wear), thereby preserving long‐term surface integrity [161].

From a temporal perspective, valve metal oxide nanostructures demonstrate predominantly inert behavior over extended durations. They exhibit exceptional stability with negligible degradation over short‐term exposure (hours to weeks) and maintain robust passive characteristics over intermediate timescales (months to years). Over long‐term periods (years to decades), gradual degradation may occur, primarily driven by synergistic effects of mechanical wear and sustained interaction with physiological fluids [174, 175, 176]. The degradation products of valve metal oxide nanostructures are predominantly minimal and chemically inert, underscoring their intrinsic high thermodynamic stability and limited solubility in physiological environments. Degradation, when it transpires—typically triggered by mechanical disruption, defect‐facilitated dissolution mechanisms, or extended exposure periods—results chiefly in the formation of ionic species, hydrated coordination complexes, and, less frequently, particulate detritus, which are chemically stable and inert [176, 177].

Overall, the toxicity and biocompatibility of valve metal oxide nanostructures are governed by material‐specific properties, dose, exposure duration, and biological context. Advances in mechanistic toxicology, toxicogenomics, and integrated in vitro/in vivo testing frameworks are essential for minimizing experimental artifacts and enabling the safe‐by‐design development of metal oxide nanomaterials for biomedical applications. Table 1 provides a broad overview of the different types of valve metal oxide nanostructures, their key properties, advantages and biomedical potential.

**TABLE 1:** Properties, advantages, and biomedical applications of valve metal oxide nanostructures.

Valve metal oxide	Key properties	Advantages	Biomedical potential/applications
TiO_2_	• Rutile, anatase, brookite phases	• Highly biocompatible	• Implant coatings
	• Chemically and thermally stable	• Strong osteointegration	• Antibacterial surfaces
	• Photocatalytic ROS generation	• Antimicrobial activity	• Drug delivery systems
	• Tunable morphology and surface area	• Tunable optical properties	• Photodynamic therapy (PDT)
	• Surface functionalization and doping possible	• Efficient drug loading	• Wound healing
		• Surface engineering capability	• Tissue engineering
			• Biosensors
			• Phototherapy and imaging
Ta_2_O_5_	• High chemical stability	• Excellent biocompatibility	• Implant and prosthetic coatings
	• Corrosion‐resistant	• Long‐term stability	• Bone tissue engineering
	• High dielectric constant	• Promotes osteoblast differentiation	• Dental tissue engineering
	• Wide bandgap	• Enhances protein adsorption	• Osteogenic scaffolds
	• High refractive index	• Reduces inflammatory responses	• Drug delivery systems
	• Amorphous or crystalline forms		
Nb_2_O_5_	• n‐type semiconductor	• Good cytocompatibility	• Implant coatings
	• Bandgap around 3.4 eV	• Enhances hydroxyapatite growth	• Wound healing
	• UV absorption capability	• Supports cell spreading	• Tissue engineering
	• Multiple structural phases	• Improves cell metabolism	• Biosensors
	• Tunable photocatalytic activity	• Tunable surface reactivity	• Potential drug delivery applications
ZrO_2_	• High hardness and toughness	• Excellent mechanical properties	• Orthopedic implants
	• Corrosion‐resistant	• Biocompatible and nontoxic	• Dental implants
	• Broad bandgap	• Supports cell growth	• Bone tissue engineering
	• High compressive strength	• Suitable for load‐bearing applications	• Antimicrobial applications
	• Radiopaque	• Fracture‐resistant	• Anticancer applications
	• Wear‐resistant		• Bone cements
HfO_2_	• Wide bandgap (5.3–5.7 eV)	• Stable in biological media	• Radiosensitizers in cancer therapy
	• High thermal stability	• Low toxicity	• Theranostic applications
	• Chemically inert	• Good cellular uptake	• Fluorescence imaging
	• High dielectric constant	• Promotes protein adsorption	• Antibacterial coatings
	• Corrosion‐resistant	• Enhances cell attachment and proliferation	• Bone and dental tissue engineering

## 8 Future Perspectives and Challenges

**FIGURE 5:**
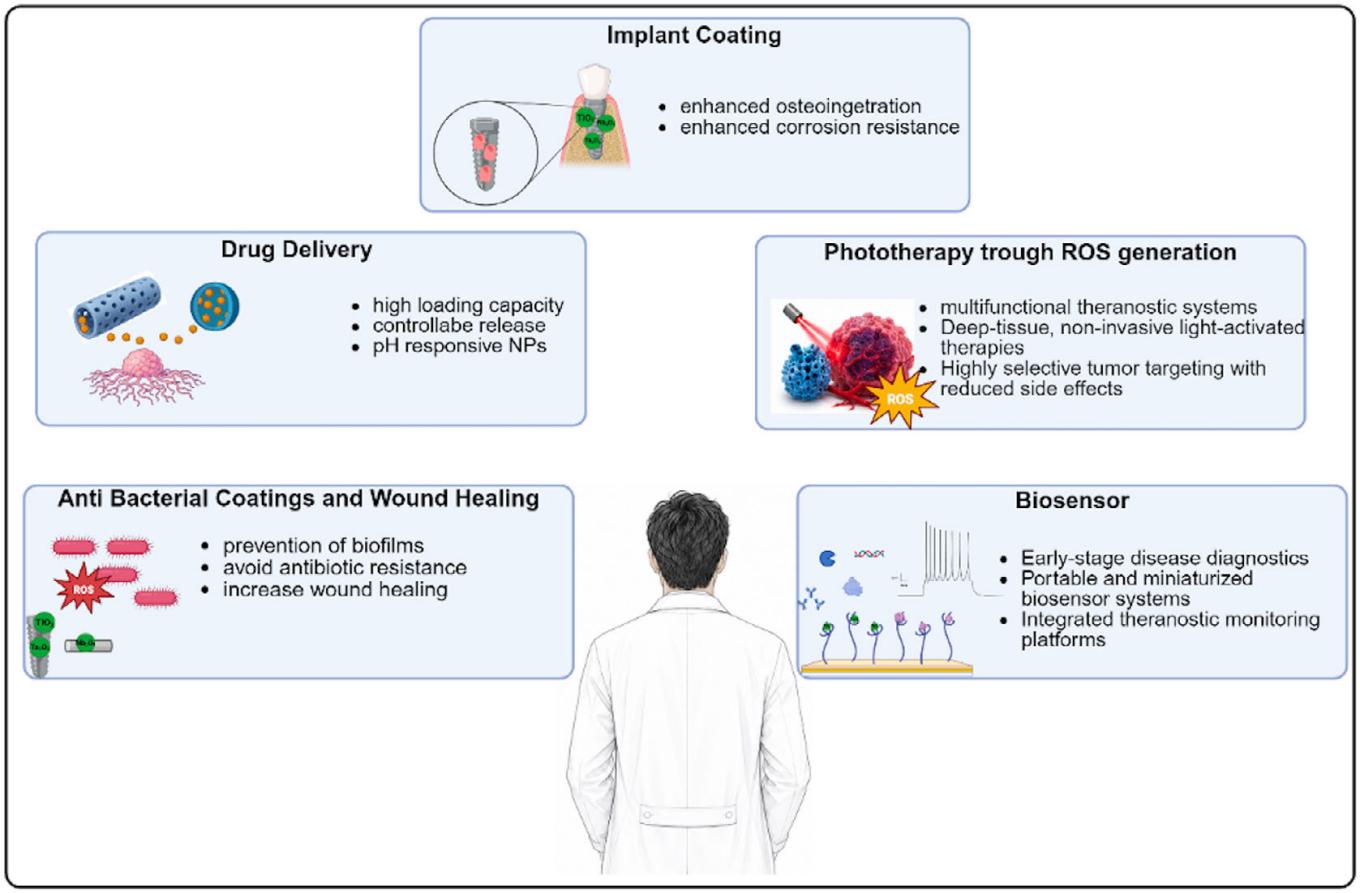
Looking ahead: Future perspectives for VMOs in biomedical applications.

Valve metal oxide nanostructures present an exquisite connection between cutting‐edge surface engineering and the forefront of biomedical advancements (Figure 5), yet their complete translational capabilities have yet to be fully harnessed. Future investigations are anticipated to concentrate on the meticulous design of surface chemistries and nanoarchitectures that facilitate precise regulation of bio‐nano interactions, encompassing protein adsorption, cellular responses, and immunomodulation. The amalgamation of multifunctional surface enhancements, such as antibacterial, osteoinductive, or drug‐eluting properties, presents a promising avenue for the development of personalized and intelligent biomedical devices. Nevertheless, considerable obstacles remain, particularly in achieving reproducible and scalable manufacturing while preserving nanoscale accuracy. Long‐term biostability, degradation characteristics, and potential ion release under physiological conditions must be rigorously assessed to guarantee safety and adherence to regulatory standards. Moreover, the absence of standardized in vitro and in vivo evaluation protocols complicates direct comparisons across studies and hinders clinical progression. Tackling these challenges through interdisciplinary collaboration, the establishment of standardized testing frameworks, and industry‐focused process optimization will be vital for effectively transitioning valve metal oxide nanostructures from theoretical surface‐engineered concepts into clinically significant biomedical technologies. Some current practices and challenges are discussed in the following sections.

### 8.1 Implant Coatings

Nanostructured metal oxide valves, particularly TiO_2_ nanotubes, have attracted considerable attention as coatings for orthopedic and dental implants [178]. The nanotubular architecture provides increased surface area for osteoblast adhesion, promoting osseointegration and improving overall implant performance. Studies have shown that TiO_2_ coatings can enhance bone growth by increasing protein adsorption, facilitating cell attachment, and accelerating healing [179].

#### 8.1.1 Challenges

1. **Mechanical stability**: Although anodized oxide layers on titanium implants are beneficial for osseointegration, these coatings often experience issues such as delamination under cyclic mechanical loading. This is particularly problematic for orthopedic implants, which are subject to significant loads over time. Adhesion between the oxide layer and the substrate can deteriorate, leading to coating failure and subsequent implant failure.
2. **Uniformity on complex geometries**: Ensuring uniform coating of three‐dimensional geometries (such as screws or trabecular structures) with nanostructures is challenging. Variations in nanotube size and surface roughness can lead to inconsistent biological responses [178, 179, 180].

### 8.2 Drug Delivery

Valve metal oxide nanostructures, particularly TiO_2_, ZnO, and ZrO_2_, are being studied for their potential for drug delivery [112, 181, 182, 183].

Nanotubes, nanopores, and mesoporous films can be loaded with therapeutic agents such as antibiotics, chemotherapeutic drugs, or growth factors. The porous structure allows for encapsulation and controlled release of drugs, while surface modifications (e.g., functionalization with polymers or biomolecules) can improve targeted delivery and reduce toxicity [113].

#### 8.2.1 Challenges

1. **Rapid release**: A significant challenge in drug delivery is the rapid release phenomenon, where a large portion of the drug is released rapidly in the initial phase. This is particularly problematic for the treatment of chronic diseases, where prolonged, controlled release is needed. This phenomenon is often a result of the surface chemistry of the nanopores and electrostatic or hydrophobic interactions between the drug and the surface.
2. **Loading capacity**: Although oxide nanostructures such as TiO_2_ are excellent for the delivery of small‐molecule drugs, their loading capacity for large biomolecules (e.g., proteins, peptides, or nucleic acids) is often limited. This is due to the small pore sizes of many oxide nanostructures, which prevent the efficient encapsulation of larger molecules.
3. **Degradation of loaded drugs**: For some oxide materials, such as TiO_2_, photocatalytic properties under UV light can lead to the degradation of sensitive drugs, limiting their use in photothermal or PDT [123, 142, 184, 185].
4. **Protein corona**: One unavoidable limitation and challenge in the biomedical application of nanoparticles is the formation of a biomolecular corona upon their exposure to biological environments. Upon entering physiological fluids such as the bloodstream or interstitial space, nanoparticles rapidly adsorb a complex layer of biomolecules, including mainly proteins but also lipids and other macromolecules, on their surface [4, 5, 163, 164]. This so‐called biocorona fundamentally alters the physicochemical properties of the nanoparticles and defines their biological identity, thereby governing their interactions with cells, biodistribution, and overall biological response [165]. The formation of a protein corona alters the originally defined physicochemical properties of nanoparticles, including, for instance, their size and surface charge. Physiologically, the corona serves as a biological interface that reduces nonspecific cellular uptake, facilitates immune recognition and clearance, and modifies the nanoparticle surface composition [4], thereby potentially masking specifically engineered antibodies and targeting ligands on the nanoparticle surface.
5. **Immune response to nanoparticles*:*** While valve metal nanoparticles are typically regarded as biocompatible and exhibit minimal intrinsic cytotoxicity, their introduction into biological systems is nonetheless perceived as a foreign stimulus, leading to activation of the immune system [166]. Several studies indicate that the introduction of, for instance, TiO_2_ Nanoparticles leads to an activation of the immune system toward a pro‐inflammatory state [167, 168, 169], which has to be considered when applying them in clinical settings. TiO_2_ nanoparticles can even lead to mitochondrial dysfunction through an increment on ROS production [167]. However, immune activation should not automatically be considered harmful and can, especially for instance in cancer therapies [169], be even exploited as an additional mechanism.

### 8.3 Antibacterial and Antiviral Surfaces

Valve metal oxide nanostructures are also being explored for antibacterial and antiviral applications. For example, TiO_2_ nanotubes exhibit excellent antimicrobial properties under UV radiation, making them ideal for wound healing and coating medical devices [98, 170].

These nanostructures can generate ROS when exposed to UV radiation, which have been shown to kill bacteria and viruses [171].

#### 8.3.1 Challenges

1. **ROS generation**: Although ROS are highly effective for sterilization and antibacterial activity, they also pose a risk of cytotoxicity to human cells. Achieving a balance between antimicrobial effect and biocompatibility is crucial, especially for medical implants and surfaces in contact with human tissue.
2. **Long‐term effectiveness**: Under physiological conditions, the antibacterial efficacy of TiO_2_ nanotubes may be reduced due to biofouling and protein corona formation. This can significantly alter their antimicrobial properties, reducing their effectiveness in real‐world conditions [98, 170, 172].

### 8.4 Biosensors

The large surface area and tunable electrochemical properties of valve metal oxide nanostructures make them excellent candidates for biosensing applications. For example, TiO_2_ and Nb_2_O_5_ nanotubes can be used in electrochemical sensors for detecting biomarkers, DNA sequences, or enzymatic reactions [15, 173, 186, 187].

#### 8.4.1 Challenges

1. **Surface functionalization**: The ability to functionalize oxide nanostructures with specific biomolecules (e.g., antibodies or aptamers) is often hampered by the surface chemistry of the oxide. Although chemical functionalization can be achieved through covalent bonding, physical adsorption, or self‐assembly, ensuring stable and reproducible functionalization is challenging.
2. **Functioning and stability**: The performance of oxide‐based biosensors can be reduced by biological fouling, in which proteins or cells adsorb to the sensor surface, altering its performance. Furthermore, maintaining long‐term stability is essential [173, 186, 187, 188].

### 8.5 Cancer Therapy and Radiation Therapy Enhancement

HfO_2_ and TiO_2_ have been studied for use in radiation therapy and cancer treatment due to their ability to absorb radiation and enhance the dose delivered to tumors. In particular, HfO_2_ has a high atomic number and is a potent radiosensitizer when incorporated into nanoparticles. These oxides can also serve as carriers for chemotherapeutic and immunotherapeutic drugs [189, 190, 191, 192].

#### 8.5.1 Challenges

1. **Toxicity and biocompatibility**: The potential cytotoxicity of radiation‐enhancing oxides such as HfO_2_ is a concern because high doses of radiation can damage surrounding healthy tissue. Furthermore, ensuring the biocompatibility of these nanoparticles is essential to minimize unwanted side effects.
2. **Size and clearance**: Nanoparticles used for radiation enhancement must be appropriately sized to ensure they reach the tumor site and avoid rapid clearance by the RES. Balancing size, surface charge, and functionalization is crucial to optimize their therapeutic potential.

## 9 Conclusion

Valve metals (TiO_2_, ZrO_2_, HfO_2_, Nb_2_O_5_, and Ta_2_O_5_, etc.) nanostructures, such as nanotubes, nanopores, nanowires, or mesoporous films, exhibit some unique properties such as high surface area, controlled porosity, and enhanced reactivity [177]. These characteristics make valve metal oxide nanostructures ideal for a variety of biomedical applications, including implant coatings, drug delivery, biosensors, antibacterial, and antiviral surfaces, and cancer therapy applications. However, the clinical application of these materials is limited by a number of issues related to biocompatibility, mechanical stability, controlled drug release, and scalability [185]. The future research topics for biomedical applications of valve metal nanoparticles are the combined approach for the construction of complex multifunctional nanosystems with their participation.

Valve metal oxide nanostructures epitomize a sophisticated and resilient category of biomaterials that seamlessly integrate surface engineering principles with the evolving needs of biomedicine. Their inherent chemical stability, resistance to corrosion, and adjustable surface chemistry—when paired with meticulous nanoscale morphological control—facilitate the precise modulation of biological interactions at the tissue–material interface. As emphasized throughout this review, oxides such as TiO_2_, Ta_2_O_5_, Nb_2_O_5_, ZrO_2_, and HfO_2_ transcend their conventional roles as mere passive implant coatings, transforming into dynamic and multifunctional platforms capable of antimicrobial activity, regulated therapeutic delivery, biosensing, and photoresponsive treatments.

In spite of these encouraging characteristics, the clinical application of valve metal oxide nanostructures is still hindered by challenges concerning long‐term biocompatibility, uncertainties in nanotoxicology, scalability of manufacturing, and the need for regulatory standardization. Overcoming these obstacles will necessitate interdisciplinary approaches that merge materials science, surface chemistry, nanofabrication, and biomedical engineering, complemented by thorough in vitro and in vivo validation processes. Specifically, future investigations should focus on the relationships between structure, properties, and biological responses, allowing for the rational design of oxide nanostructures tailored to particular medical applications.

Looking ahead, the fusion of advanced surface functionalization, stimuli‐responsive design, and bioinspired engineering is anticipated to unleash the full potential of VMOs as next‐generation biomedical materials. By evolving from inert substrates to dynamically interactive interfaces, these nanostructures promise to significantly influence the development of innovative, safer, and more effective biomedical technologies across the realms of regenerative medicine, diagnostics, and targeted therapies.

## Author Contributions

**Nina** **Kummer**, **Désirée Gül**, **İdris Sargin**, **Mustafa Dolaz**, **Dzmitry Shcharbin**, **Burcu Önal Acet**, **Emrah Dikici**, **Mingyuan Gao**, **Mehmet Odabaşı**: conceptualization, writing – original draft preparation, investigation, writing – review and editing. **Shirley. K. Knauer**: writing – review and editing. **Roland H. Stauber**: writing – original draft preparation, writing – review and editing, funding. **Ömür Acet**: conceptualization, writing – original draft preparation, investigation, writing – review and editing, supervision. All authors have read and agreed to the published version of the manuscript.

## Conflicts of Interest

The authors declare no conflicts of interest.

## Data Availability

Data sharing not applicable to this article as no datasets were generated or analysed during the current study.
